# Stimuli-Responsive Starch-Based Biopolymer Coatings for Smart and Sustainable Fertilizers

**DOI:** 10.3390/gels11090681

**Published:** 2025-08-26

**Authors:** Babar Azeem

**Affiliations:** Department of Chemical Engineering, College of Engineering, Imam Mohammad Ibn Saud Islamic University (IMSIU), Riyadh 11432, Saudi Arabia; badin@imamu.edu.sa

**Keywords:** starch-based biopolymers, controlled-release fertilizers, stimuli-responsive polymers, biodegradable coatings, smart nutrient delivery, sustainable agro-technologies

## Abstract

The quest for sustainable agriculture demands nutrient delivery systems that align productivity with environmental responsibility. This review critically evaluates stimuli-responsive starch-based biopolymer coatings for controlled-release fertilizers (CRFs), highlighting their structure, functionality, and agronomic relevance. Starch, an abundant and biodegradable polysaccharide, offers intrinsic advantages such as modifiability, film-forming ability, and compatibility with green chemistry. The paper discusses starch’s physicochemical characteristics, its functionalization to achieve responsiveness to environmental triggers (pH, moisture, temperature, ionic strength), and coating strategies like in situ polymerization, grafting, and nanocomposite integration. A comprehensive analysis of release kinetics, swelling behavior, biodegradability, and water retention is provided, followed by evaluations under simulated field conditions, encompassing various soil types, environmental stressors, and crop responses. Comparative insights with other smart biopolymers such as chitosan, alginate, and cellulose underscore starch’s unique position in CRF technology. Despite promising developments, the review identifies critical research gaps, including limitations in scalability, coordination of multi-stimuli responses, and the need for extensive field validation. This work serves as a consolidated platform for researchers, policy makers, and agro-industrial stakeholders aiming to design smart, eco-friendly fertilizers that address global food security while minimizing ecological footprints.

## 1. Introduction

The global demand for sustainable and resource-efficient agriculture has accelerated the innovation of environmentally responsive nutrient delivery systems [1]. Traditional fertilizers suffer from poor nutrient use efficiency, often leading to nutrient leaching, volatilization, and waterway eutrophication. In this context, CRFs have emerged as promising tools to synchronize nutrient release with crop uptake and reduce environmental impact [2].

Among CRFs, starch-based materials have attracted increasing attention due to their biodegradability, renewability, cost-effectiveness, and film-forming properties [3]. However, native starch often lacks the robustness required for field applications, necessitating structural modifications and reinforcements [4]. The development of hybrid coating systems that incorporate stimuli-responsive polymers and nanomaterials into starch matrices has addressed these limitations, enabling precise nutrient release under environmental triggers such as pH, temperature, and soil moisture [5].

Stimuli-responsive systems are designed to respond dynamically to changes in environmental conditions, allowing for “smart” fertilizer formulations that release nutrients in a controlled and targeted manner [6]. For example, pH-sensitive coatings degrade faster in acidic soils, while moisture-sensitive hydrogels expand and release nutrients during irrigation or rainfall events. Such responsiveness not only enhances nutrient availability but also contributes to soil health and reduced input requirements [7].

In recent years, several approaches have been explored to enhance the performance of starch-based CRFs, including chemical grafting, nanofiller reinforcement, polyelectrolyte multilayers, and encapsulation in hydrogel beads. These developments have broadened the functional landscape of starch-based fertilizers, making them viable for precision agriculture and sustainable farming systems [8,9].

Despite the progress, comprehensive reviews integrating the chemistry, material design, fabrication techniques, and performance metrics of such smart starch-based CRFs remain limited. This review addresses this gap by focusing on recent advances in the development of stimuli-responsive starch-based hybrid coatings. It critically evaluates synthesis methods, responsiveness mechanisms, structural–functional correlations, and agronomic performance under field-relevant conditions. The goal is to provide a consolidated framework for future innovation and application of smart fertilizer delivery systems aligned with sustainable development goals (SDGs).

## 2. Methodology

### 2.1. Methodological Framework for Literature Selection and Analysis

To ensure a comprehensive and objective synthesis of the literature on starch-based CRFs, this review adopted a hybrid methodological framework that integrates elements of both scoping reviews and systematic literature reviews. The strategy aligns with PRISMA-ScR guidelines for scoping reviews and follows the structured review pathways outlined in the recent methodological literature [10].

First, a scoping review approach was adopted to map the breadth and diversity of the literature and identify relevant research trends, key materials (especially starch and its derivatives), coating strategies, release mechanisms, and stimuli-responsive systems. This method is ideal for exploring broad topics with heterogeneous evidence and aligns with the typology proposed by Grant and Booth, which distinguishes between scoping, mapping, and narrative reviews [11].

The literature search process covered multiple databases including Scopus, Web of Science, and Google Scholar, targeting peer-reviewed articles published primarily from 2020 to 2025. Keywords included combinations of terms such as “starch-based hydrogel”, “controlled-release fertilizer”, “stimuli-responsive coating”, “biopolymer encapsulation”, “CRF kinetics”, and “environmentally responsive fertilizers”. Boolean operators and truncations were used to maximize retrieval [12].

Following the scoping stage, systematic screening and eligibility assessment were carried out using PRISMA-based flow protocols [13]. Articles were included based on their relevance to starch-based materials in CRF applications, with exclusion criteria applied to eliminate irrelevant, duplicate, or non-peer-reviewed content. A total of 168 articles were shortlisted for full-text review and critical appraisal.

To structure the review further and ensure balanced topic coverage, the 10-step bibliometric–systematic protocol proposed by Almozayen et al. [14] was followed. This allowed thematic clustering based on shared concepts such as material synthesis, performance evaluation, release behavior, and environmental impact. Key performance metrics were extracted and organized under conceptual categories aligned with the review objectives.

In parallel, data mining techniques were used to explore co-occurrence of keywords and co-authorship networks, identifying research hotspots and collaboration patterns in the field of smart CRF development [15]. This analytical approach enabled evidence-based categorization of technological progressions and material innovations, particularly around starch grafting, nanocomposite hydrogels, and stimuli-triggered nutrient delivery.

This methodological design ensures that the review is both comprehensive in coverage and analytically robust, offering clarity on where the field has been and where it is headed. It also enhances transparency and reproducibility, two critical pillars of modern scientific review practice [12].

### 2.2. Inclusion and Exclusion Criteria

A well-defined set of inclusion and exclusion criteria is vital to ensure transparency, reproducibility, and methodological rigor in systematic or structured reviews. For this review, the selection of sources was driven by both relevance to the core themes, namely starch-based coatings, stimuli-responsive delivery systems, and their role in CRFs; and adherence to scholarly and technical quality standards.

Inclusion criteria were informed by recommendations in systematic review methodologies for evidence-based research synthesis [16,17]. Specifically, studies were included if they

- Reported original experimental or review data on starch-based or polysaccharide-modified coatings applied in CRF systems;
- Focused on hydrogel design, smart release mechanisms (e.g., moisture, pH, or temperature responsiveness), or nanomaterial-enhanced coatings;
- Were published in peer-reviewed journals from 2020 onward;
- Were written in English;
- Provided accessible full-text articles (Open Access/Subscription).

To maintain relevance and avoid overgeneralization, exclusion criteria eliminated studies that

- Focused solely on drug delivery or pharmaceutical encapsulation without agrochemical application;
- Addressed fertilizer efficiency without discussing coating mechanisms or polymer matrix design;
- Were editorials, abstracts without full text, or lacked methodological clarity.

The process for defining these criteria aligns with the principles described by Booth et al. [18] and the typology of reviews discussed by Grant and Booth [19], which emphasize aligning review scope with intended outcomes and user needs. The selection framework also took guidance from the work of Meline [20], emphasizing the need to document how decisions about article inclusion or exclusion are justified with reference to research objectives.

By clearly articulating these boundaries, this review ensures that the collected literature meaningfully contributes to the design, evaluation, and application of starch-based smart fertilizers, reflecting both the scientific advances and practical challenges in the field.

### 2.3. Bibliometric and Publication Trends

The evolution of starch-based CRFs, especially those enhanced with stimuli-responsive and nanocomposite coatings, reflects a growing research focus on sustainable agriculture, precision nutrient delivery, and biodegradable materials [21]. Bibliometric analysis provides a macro-level overview of the knowledge structure and development trajectory of this interdisciplinary field.

In the past two decades, there has been a marked increase in publications relating to starch-based CRFs, particularly after 2010, driven by global concerns regarding nutrient losses, environmental degradation, and the circular economy. An initial search in the Scopus database using keywords such as “starch-based fertilizer,” yielded 541 peer-reviewed articles published between 2000 and 2025. Of these, approximately 67% were published in the last five years, indicating a sharp upward trend in research interest. The majority of contributions are from China, India, the USA, Brazil, and Iran; regions facing high agricultural demands and environmental pressures.

**Figure 1:**
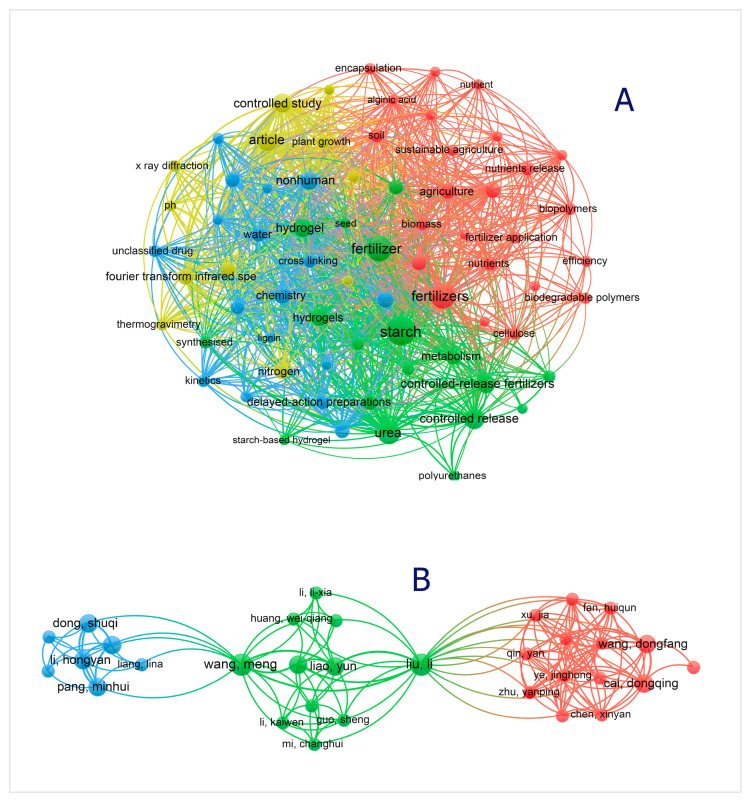
Bibliometric visualization of (**A**) keyword co-occurrence and (**B**) author collaboration in the field of starch-based CRFs (2005–2025).

Journals leading in this area include *Carbohydrate Polymers*, *Journal of Agricultural and Food Chemistry*, *ACS Sustainable Chemistry & Engineering*, and *Journal of Controlled Release*. Furthermore, the collaboration networks show increasing international partnerships between agricultural institutes and materials science laboratories, reflecting the field’s inherently interdisciplinary nature (see Figure 1).

In terms of thematic focus, the literature reveals a transition from basic formulation of CRFs to more advanced systems integrating pH-/moisture-responsive polymers, hydrogel matrices, nanofillers like biochar, SiO_2_, and zeolites, and sustainability-oriented performance metrics. Keyword co-occurrence analysis highlights dominant clusters around terms such as “starch” “fertilizers,” “controlled-release fertilizers,” “urea,” and “hydrogel.” This evidences a paradigm shift from merely slowing nutrient release to engineering multifunctional delivery platforms (Figure 1A).

The bibliometric landscape illustrates a rapidly maturing domain, with starch-based smart fertilizer coatings situated at the intersection of green chemistry, agricultural biotechnology, and polymer materials science. Figure 1B indicates the co-authorship network related to the research conducted for stimuli-responsive starch biopolymer-based slow/controlled-release fertilizers.

## 3. Starch as a Functional Biopolymer for Coated Fertilizers

In the search for sustainable and intelligent CRFs, the structural tunability of starch allows for diverse chemical modifications, enabling tailored nutrient release profiles under variable environmental conditions [22,23]. This section critically examines starch from three complementary perspectives: its physicochemical characteristics, the spectrum of chemical and physical modifications employed to enhance its performance, and its emerging role as a smart material responsive to environmental stimuli. Together, these insights underscore starch’s unique position in the design of advanced coating systems for next-generation fertilizers.

### 3.1. Structural and Physicochemical Characteristics of Starch

Structurally, starch comprises two major glucose-based polymers: amylose (a mostly linear α-1,4-glucan) and amylopectin (a highly branched α-1,4 and α-1,6-glucan) [24,25]. The relative proportion of these polymers significantly affects starch’s physicochemical behavior, including gelatinization, retrogradation, water solubility, and film-forming ability [26,27].

Amylose typically constitutes 20–30% of native starch, whereas amylopectin accounts for the remaining 70–80%. High amylose content improves film integrity and tensile strength but reduces water solubility and swelling, traits beneficial for prolonged nutrient release in coated fertilizers [28,29]. Amylopectin-rich starches, on the other hand, exhibit enhanced water uptake, gelatinization, and viscosity, favorable for hydrophilic delivery matrices but less desirable for applications requiring moisture barrier properties [27,30].

Granule morphology of starches varies with botanical origin and processing. Typically, native starch granules are semi-crystalline, comprising alternating amorphous and crystalline lamellae (9–10 nm repeat distance). X-ray diffraction reveals three principal types of crystallinity: A-type (cereals), B-type (tubers), and C-type (legumes), each affecting hydration, digestibility, and processing response [26,31].

Starch’s thermal behavior is characterized by gelatinization; a phase transition during heating in the presence of water. Gelatinization temperature and enthalpy are influenced by crystallinity, moisture content, and amylose-to-amylopectin ratio. These properties determine starch’s suitability for spray-coating and encapsulation processes, where thermal and mechanical stability are critical [27,32].

Chemically, starch exhibits abundant hydroxyl groups that facilitate physical interactions (e.g., hydrogen bonding) and chemical modifications (e.g., grafting, crosslinking). This tunability underpins starch’s widespread utility in functional biopolymer systems. For instance, its water sensitivity and biodegradability can be tailored by altering chain mobility or crystallinity or substituting hydroxyl groups with hydrophobic or ionic moieties [33,34].

Together, these structural and physicochemical properties make starch a dynamic, multifunctional platform for developing environmentally benign CRF coatings. The adaptability of starch allows its integration into hybrid or composite systems with tunable release profiles responsive to soil moisture, pH, or microbial activity, contributing to smart, climate-resilient agriculture [26,34].

**Figure 2:**
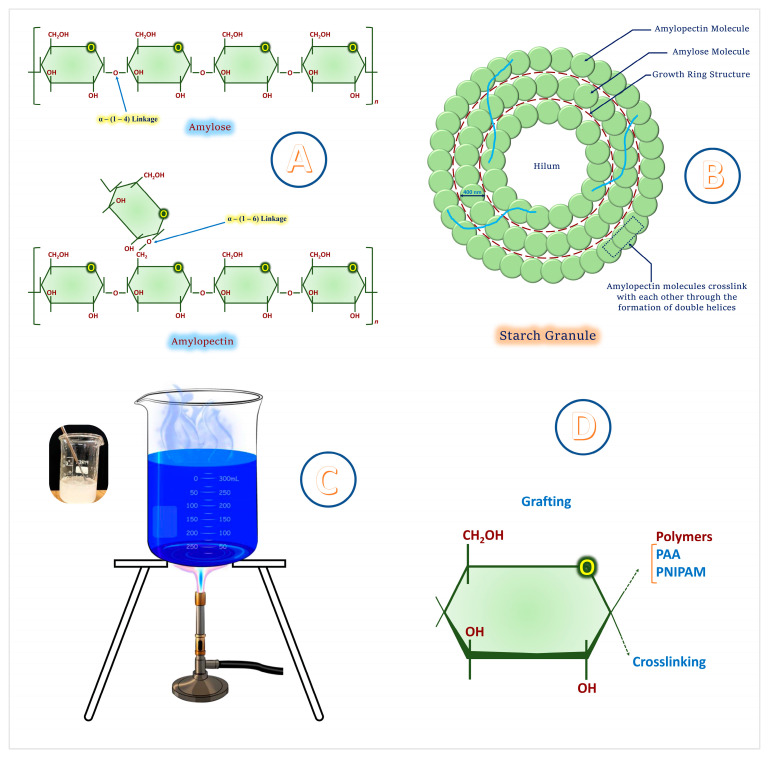
(**A**) Molecular structure of starch. (**B**) Native starch granule morphology. (**C**) Thermal and film-forming properties depicting gelatinization in the 80–95 °C range. (**D**) Functional sites on the starch backbone.

The unique structural and functional attributes of starch, including its dual-polymer composition, semi-crystalline granule morphology, thermal behavior, and modifiable hydroxyl groups, collectively enable its effective use in CRF coatings (Figure 2).

### 3.2. Modification Techniques and Functionalization Strategies

Native starch suffers from inherent limitations such as water sensitivity, low mechanical strength, and poor thermal stability, which restrict its direct application in CRF coatings. Therefore, various modification techniques have been developed to tailor its physicochemical properties and enable functional responsiveness to environmental stimuli [35].

Chemical modifications such as grafting, crosslinking, and oxidation are among the most widely explored strategies. Grafting synthetic or natural polymers onto starch chains, via free radical polymerization or enzymatic catalysis, alters hydrophilicity, mechanical properties, and biodegradability. For instance, grafting vinyl monomers like poly(acrylic acid) or poly(vinyl acetate) has been shown to improve water absorption and nutrient retention characteristics [4]. Channab et al. (2024) demonstrated that starch-g-poly(vinyl acetate) significantly slowed nitrogen release from urea granules due to enhanced barrier properties [36].

Crosslinking using agents such as glutaraldehyde, epichlorohydrin, or citric acid can reinforce the starch structure by forming covalent interchain bridges, thereby improving thermal stability and decreasing solubility. As reported by Song et al. (2024), citric acid-crosslinked starch films exhibit improved dimensional stability and water resistance, which are desirable in CRF matrices [37].

In addition to conventional chemical modification, green and physical strategies like microwave-assisted esterification, enzymatic modification, or ultrasonication are gaining traction due to their energy efficiency and environmental safety [38]. These methods offer fine control over substitution degrees and surface functionalization, which are essential for tuning response kinetics under varying soil conditions.

Functionalization with smart moieties, such as temperature- or pH-sensitive groups, adds a stimulus-responsive dimension to starch matrices. Incorporation of components like poly(N-isopropylacrylamide), carboxymethyl cellulose, or polydopamine creates starch-based matrices capable of adjusting permeability or degradation rates in response to environmental triggers; an attribute critical for precision agriculture [39].

Furthermore, blending strategies have also proven effective in enhancing the functionality of starch matrices. Hybrid films formed by blending starch with biopolymers (e.g., chitosan or alginate) or nanomaterials (e.g., cellulose nanocrystals or montmorillonite) not only improve barrier and release properties but also impart antibacterial or moisture retention functions, expanding their agricultural utility [40].

The versatility of starch as a base material lies in the wide range of functionalization routes available. These enable the design of sustainable and responsive CRF systems tailored to specific agronomic and environmental needs.

### 3.3. Stimuli-Responsive Properties of Starch-Based Coatings: Mechanisms and Applications

Stimuli-responsive polymers, often termed “smart polymers,” exhibit reversible physicochemical changes when exposed to slight environmental variations such as pH, temperature, moisture, enzymatic activity, or ionic strength [41]. Modified starch, through grafting, blending, or chemical functionalization, demonstrates such responsive behaviors and serves as an excellent platform for CRF coatings [42]. These responsive mechanisms enable the fertilizer to align nutrient release with crop uptake requirements under fluctuating soil conditions.

#### 3.3.1. pH-Responsive Behavior

pH-responsive starch-based systems rely on the presence of ionizable functional groups within the polymer network, such as carboxyl or amino moieties, which undergo protonation or deprotonation depending on the surrounding pH [43]. Under acidic conditions, protonation reduces electrostatic repulsion between polymer chains, resulting in a denser structure with lower permeability. In contrast, under alkaline conditions, deprotonation increases electrostatic repulsion and generates osmotic pressure from counterions, together leading to polymer swelling, increased pore size, and enhanced nutrient diffusion [44]. Such behavior enables the modulation of nutrient release rates in response to soil pH fluctuations, aligning nutrient availability with plant uptake requirements [45]. Chemical modifications, such as grafting acrylic acid, introducing anionic groups, or blending with other pH-sensitive polymers, have been shown to fine-tune swelling behavior and release kinetics [43,44].

A Schiff-base crosslinked CMS hydrogel, for example, demonstrated significantly faster urea release at pH 7.4 compared to pH 4.0, owing to higher ionization and increased water uptake [46]. This indicates that pH governs the polymer’s permeability and nutrient diffusion characteristics.

Hydrogen bonding and electrostatic interactions further modulate this response. At low pH, intra- and intermolecular hydrogen bonding compacts the polymer network, whereas at high pH, these interactions are disrupted, promoting gel expansion and nutrient mobility [47]. This gel–sol transition behavior is common in responsive biopolymer systems and directly affects release timing and rate.

Crosslinking agents like Ca^2+^ and citric acid also play critical roles. In starch–alginate matrices, Ca^2+^ ions form “egg-box” junctions that hold the network tightly in mildly acidic-to-neutral conditions but swell or partially dissociate in basic media due to chelation effects [47]. This enables pH-sensitive permeability and sustained release.

In another study, oxidized starch hydrogels showed enhanced nutrient release in alkaline environments, attributed to increased hydrophilicity and reduced network rigidity [48]. Notably, the crosslinking density and type of functionalization (carboxylation, oxidation) significantly influence pH sensitivity and nutrient transport properties.

Many pH-responsive starch-based hydrogels also exhibit Fickian diffusion behavior, where nutrient release is directly tied to matrix swelling and relaxation time [48]. Some formulations demonstrate adjustable release durations ranging from 7 to 30 days, depending on pH-triggered structural transitions [49].

Conclusively, the molecular mechanisms underpinning pH responsiveness in starch-based CRFs involve [50]

- Ionization of functional groups (–COOH ⇌ –COO^−^);
- Hydration-induced expansion or collapse of the matrix;
- Hydrogen bond formation/disruption;
- pH-sensitive ionic crosslink stability (e.g., Ca^2+^, STMP);
- Matrix relaxation that modulates diffusion pathways.

These features enable starch-based coatings to function as intelligent delivery systems, aligning nutrient release with environmental cues and minimizing wasteful leaching under variable pH conditions.

#### 3.3.2. Temperature-Responsive Behavior

Thermoresponsive hydrogels based on starch have been developed by incorporating temperature-sensitive moieties such as poly(N-isopropylacrylamide) (PNIPAM). Below the lower critical solution temperature (LCST), these hydrogels remain swollen, encapsulating nutrients. Above the LCST, they shrink and release their content due to expulsion of water [51,52]. This temperature-triggered mechanism aligns nutrient availability with growing season temperature profiles, offering benefits for crops under fluctuating climates [53,54].

For instance, in a starch–PNIPAM composite reported by Balçık Tamer et al. [55], the LCST behavior enabled nitrate release to accelerate during cooler periods, aligning with plant nutrient uptake cycles. Similar results were obtained in a polyacrylamide–starch hydrogel, where the degree of swelling dropped significantly when the temperature exceeded 35 °C, reducing nutrient efflux [56].

In another case, a thermoresponsive hydrogel synthesized from carboxymethyl cellulose-stabilized N-vinylcaprolactam (CMC–NVCL) demonstrated precise thermal control of release. This system exploited the temperature-dependent coil–globule transition of NVCL moieties and was shown to release urea more rapidly at 25 °C than at 37 °C, a reversal of typical behavior and useful in subtropical agriculture [57].

Mechanistically, temperature elevation causes dehydration of hydrophilic groups and increases chain mobility, facilitating polymer matrix contraction. This leads to decreased mesh size and lower nutrient diffusion. In systems incorporating ionic crosslinks or nanofillers, the responsiveness can be further tuned. For example, Xu et al. [58] used ionic crosslinked starch-based hydrogels with enhanced thermal responsiveness via calcium-induced coordination chemistry.

Notably, dual-responsive systems, reacting to both pH and temperature, allow multi-environmental control over release. Feng et al. [54] demonstrated that nutrient release in a polydopamine-grafted hydrogel was significantly accelerated under low temperature and high pH, but suppressed under high temperature, suggesting the interplay of polymer brush hydration and ionic dissociation.

Moreover, a methylcellulose-based hydrogel with embedded K_2_SO_4_ showed tunable gelation temperature and modulated release behavior. Incorporation of salts acted as ionic plasticizers, adjusting both mechanical strength and transition temperature, a strategy transferrable to starch systems [59,60].

At the molecular level, thermoresponsiveness in starch composites arises from

- Reversible disruption of intermolecular hydrogen bonds between hydroxyl groups;
- Temperature-sensitive hydrophobic interactions among grafted or blended moieties;
- Coil-to-globule transitions in synthetic grafts like PNIPAM or NVCL;
- Crosslinking density and type (e.g., ionic vs. covalent) affecting flexibility and collapse.

**Figure 3:**
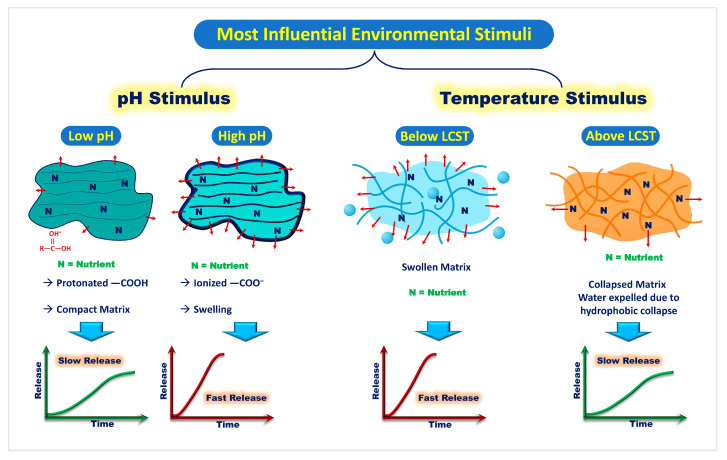
Molecular mechanisms of pH and temperature responsiveness in modified starch-based coatings.

Despite these advantages, challenges remain. Excessive crosslinking can hinder responsiveness, while phase transitions may cause structural fatigue over multiple cycles. Therefore, optimal formulation must balance mechanical stability, LCST tuning, and biodegradability. The ionization and phase transition behaviors of modified starch hydrogels enable precise nutrient release under variable pH and temperature conditions, as illustrated in Figure 3.

#### 3.3.3. Moisture-Responsive Behavior

Water-sensitive starch-based hydrogels swell in humid or irrigated conditions, enabling nutrient release, and contract during dry periods to prevent loss. This moisture-controlled behavior is one of the most common stimuli for smart fertilizers. Studies show that such hydrogels can double as water reservoirs while gradually releasing nutrients, enhancing water use efficiency [61,62].

Moisture-responsive CRFs are typically developed using hydrophilic starch-based hydrogels and superabsorbent polymers (SAPs) that swell in the presence of water. This swelling mechanism controls the diffusion of encapsulated nutrients, making it possible to synchronize release patterns with plant water uptake cycles. The molecular basis of this behavior lies in the hydrophilic groups (e.g., hydroxyl, carboxyl, and amide groups) grafted onto the starch backbone. These groups ionize in the presence of water, generating mobile counterions within the polymer network. The osmotic pressure arising from these counterions, combined with electrostatic repulsion between like-charged groups, drives water uptake and swelling of the hydrogel network [63,64].

For example, Song et al. [65] developed eco-friendly starch-based SAPs grafted with acrylamide and acrylic acid, which exhibited high swelling capacity and moisture retention. This not only enhanced water availability in the rhizosphere but also regulated the release of nitrogen fertilizers through a diffusion-driven mechanism. Similarly, Agbna and Zaidi [66] emphasized that hydrogels enhance plant resilience under water-deficient conditions by maintaining consistent moisture levels, thus influencing nutrient diffusion and bioavailability.

Tiamwong et al. [47] proposed a starch–alginate composite hydrogel system that demonstrated low responsiveness to excess humidity but retained efficient release under moderate moisture, reducing leaching risk. This tunable sensitivity makes such systems valuable for diverse agroecological zones. Likewise, a composite system integrating starch and microalgae-based hydrogels reported by Sarhan et al. [67] showed high re-swelling capacity and moisture-triggered release of encapsulated urea.

In another study, a cellulose–starch blend was shown to have enhanced structural stability and swelling ratio, improving its effectiveness as a moisture-responsive matrix for nutrient delivery [68]. Such materials offer not only gradual release but also potential for soil conditioning and reduced water stress.

In addition, the degree of crosslinking and the type of monomer grafted significantly impact the water absorption profile and hence the moisture responsiveness of the final material. High crosslinking densities, while improving mechanical stability, may reduce water uptake and slow the swelling-induced release, whereas lower densities result in faster nutrient diffusion but at the cost of structural integrity [69].

Moisture-responsive CRFs also contribute to sustainability by reducing irrigation needs and minimizing nutrient losses during periods of heavy rainfall. As reported by Ma et al. [65], starch-based hydrogels not only improved water use efficiency but also mitigated nutrient leaching during heavy rainfall events by absorbing excess water, swelling to entrap dissolved nutrients, and releasing them gradually as soil moisture decreases. While water availability is a major factor in nutrient release, soil salinity and ionic strength further complicate swelling behavior and must be considered in CRF design.

#### 3.3.4. Enzyme-Responsive Behavior

Soil microbiota secrete enzymes like amylase and cellulase that can degrade polysaccharide coatings. These enzymes are often localized in the rhizosphere, where microbial activity is highest, leading to preferential degradation of the coating near active root zones [70]. This spatial targeting enables nutrients to be released primarily in regions where plant uptake is maximized, minimizing losses through leaching or volatilization. The specificity arises from matching the coating’s chemical linkages, such as α-1,4-glycosidic bonds in starch or ester linkages in modified polysaccharides, to the enzymatic profile of the target soil microbiota [71]. For example, starch–PVA matrices were found to break down more rapidly in microbially rich rhizosphere soils than in bulk soil, allowing nutrient release to align closely with root-zone biology [72].

Starch, due to its biodegradable α-1,4- and α-1,6-glycosidic linkages, is particularly susceptible to microbial enzymatic attack in soil environments. Soil microorganisms, especially Arthrobacter, Pseudomonas, and Bacillus species, are known to secrete extracellular amylases that hydrolyze starch into glucose and oligosaccharides. For example, a study reported that the degradation of a corn starch–sodium alginate-based film led to a substantial restructuring of the soil microbial community. After 14 days, Arthrobacter became the dominant genus with a 16.48-fold increase, while Acidophilus levels dropped drastically [73].

This microbial succession is closely related to the presence of biodegradable polysaccharides in the matrix. The early stage of degradation is often marked by a bloom of fungal genera such as Alternaria and Cladosporium, which produce hydrolytic enzymes facilitating the breakdown of complex polysaccharides [74].

As the starch-based coatings degrade, nutrient release becomes increasingly governed by microbial activity. The porosity of the degraded structure increases due to enzymatic etching and microbial tunneling, allowing for diffusion-controlled release. Santos et al. (2025) demonstrated that enzyme-triggered degradation could synchronize nutrient availability with root demand, particularly under moist and warm soil conditions optimal for microbial metabolism [75].

Furthermore, enzymatic specificity plays a role in the timing of nutrient delivery. Amylases degrade linear and branched starch components at different rates, resulting in a staggered release profile. When combined with other natural polymers like sodium alginate or cellulose, the degradation becomes more tunable. For instance, the presence of pectinases and cellulases in the soil further modifies the matrix porosity and strength [76].

The biodegradation of starch-based CRFs does not merely influence nutrient kinetics; it also positively impacts soil health. This gradual decomposition enriches soil organic matter and promotes microbial diversity. Mulch degradation increased soil organic matter and led to the proliferation of beneficial microbial consortia. Additionally, an increase in fungal biomass, especially from Ascomycota, was noted, suggesting robust microbial turnover during the degradation phase [73].

Importantly, enzymatic degradation does not lead to nutrient leaching, as the slow and biologically mediated release aligns better with crop uptake, minimizing environmental losses. This responsiveness to soil microbiota is a major advantage over conventional CRFs.

Despite the promising potential of microbial and enzymatic responsiveness, several challenges remain. Soil microbial activity is highly variable and influenced by pH, temperature, and moisture. Ensuring consistent performance across diverse agro-climatic zones is difficult. There is also a need for more targeted design of CRF coatings that are selectively degradable by beneficial microbes, without encouraging pathogenic strains [77].

Recent advances have suggested that bioengineering microbial consortia or incorporating prebiotic agents into the fertilizer matrix could enhance site-specific degradation and nutrient delivery. This opens pathways for microbe-responsive fertilizers that adapt to specific soil microbiomes.

#### 3.3.5. Ionic Strength and Electrolyte Sensitivity

Starch-based polyelectrolyte complexes (PECs), formed by blending with alginate or chitosan, demonstrate sensitivity to ionic strength changes in soil. Elevated salt concentrations compress the double layer, reducing electrostatic repulsion and causing polymer collapse. This property was exploited in coatings that de-swell in saline conditions, slowing nutrient diffusion and avoiding leaching [78].

Hydrogels swell primarily due to the osmotic pressure difference between the internal polymer network and the external solution. When the ionic strength of the surrounding medium increases, the osmotic pressure gradient decreases due to the shielding of charged groups in the polymer network. This phenomenon, referred to as the Donnan effect, leads to reduced swelling as external cations neutralize the negatively charged sites within the hydrogel, especially carboxylate or phosphate groups introduced through grafting or chemical modifications [79].

In the context of agriculture, soils with varying salinity levels (e.g., saline–alkali soils or regions using saline water for irrigation) significantly affect the performance of starch-based CRFs. For example, a study demonstrated that ionic crosslinking of starch with iron ions not only improved structural stability but also reduced the hydrogel’s responsiveness in high-ionic-strength media, thereby slowing nutrient diffusion [58]. Similarly, other researchers have shown that the release rate of nitrogen from ionic hydrogels slows down in saline solutions due to the suppressed water absorption and reduced polymer chain mobility [47,49].

Moreover, crosslinked networks containing ionizable functional groups such as –COOH or –SO_3_H exhibit strong sensitivity to ionic environments. In saline conditions, these groups can exist in deprotonated form (–COO^−^, –SO_3_^−^), making them electrostatically attractive to cations in solution. Monovalent cations (Na^+^, K^+^) can partially shield the electrostatic repulsion between adjacent polymer chains, causing network contraction, while divalent cations (Ca^2+^, Mg^2+^) can bridge two negatively charged sites on different chains, inducing ionic crosslinking. This ion-exchange process displaces bound H^+^ ions without necessarily lowering bulk pH, as protons may remain solvated or buffered in the medium. The resulting chain contraction or ionic crosslinking reduces free volume, limits water penetration, and causes hydrogel collapse, leading to sharp decreases in swelling and nutrient release behavior [80,81].

A study by Simão et al. (2024) highlighted the ionic strength-dependent behavior of starch-based hydrogels used in sandy and clay soils. In low-ionic-strength conditions, water uptake was up to five times greater than in saline conditions, indicating the crucial role of soil composition in the performance of such hydrogels [82]. These findings underscore the need for careful formulation of CRFs tailored to the ionic characteristics of the soil where they are to be applied.

Efforts to mitigate ionic sensitivity have included the incorporation of non-ionic co-monomers or layered coatings that act as semi-permeable membranes. For instance, multi-layered or amphiphilic coatings incorporating starch with neutral polymers like polyvinyl alcohol have been reported to buffer the ionic interaction, maintaining nutrient release even under fluctuating salt concentrations [48,50].

The ionic strength of the external environment is a significant variable that governs the structural dynamics and release characteristics of starch-based CRFs. An in-depth understanding of these interactions allows for better design of fertilizer formulations suited for saline-prone regions, contributing to improved nutrient use efficiency and reduced environmental impact [83].

**Figure 4:**
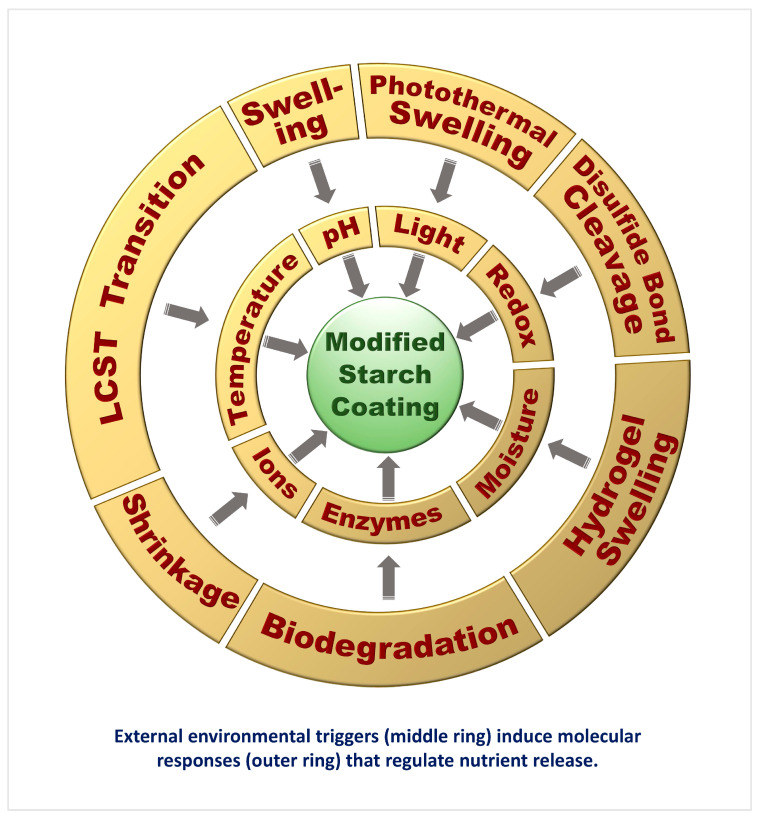
Summary of environmental stimuli and their responsive mechanisms in modified starch-based fertilizer coatings.

These diverse environmental triggers and their associated molecular mechanisms are collectively illustrated in Figure 4.

#### 3.3.6. Light Responsive Mechanisms

Light-responsive controlled-release systems represent an emerging frontier in smart agrochemical delivery, offering spatial and temporal precision in nutrient release. Although phototriggered systems are more commonly associated with biomedical applications, recent advances have demonstrated their promising potential in sustainable agriculture. In the context of starch-based CRFs, photothermal and photoisomerization mechanisms have been leveraged to modulate structural dynamics and nutrient diffusion.

Among these, photothermal-responsive systems are the most relevant to starch matrices. For instance, Li et al. developed a sodium alginate/carboxymethyl starch/polydopamine (PDA)-based CRF that utilizes PDA’s light-absorbing capacity to facilitate photothermal conversion under solar irradiation [84]. The generated heat softens or swells the hydrogel matrix, enabling faster urea release, particularly under strong light conditions. The study demonstrated that PDA significantly enhances plant growth, root development, and stress resistance while contributing to the dynamic release triggered by light exposure.

This light-mediated control is particularly effective when integrated with biopolymers like carboxymethyl starch (CMS), which undergo hydration and chain mobility changes upon mild heating. As the matrix absorbs light and converts it to thermal energy, the localized heating can increase the mobility of CMS chains, causing swelling and increased porosity. This, in turn, promotes nutrient diffusion, as seen in light-responsive alginate–starch composites [84].

Beyond photothermal effects, light-sensitive chemical bonds, such as azobenzene or o-nitrobenzyl groups, have been used to create UV-cleavable linkages in starch-derived hydrogels [48]. However, these strategies are still rare in fertilizer formulations due to concerns over material toxicity and limited sunlight penetration in soil environments. Yet, their successful implementation in other fields opens avenues for future innovation in environmentally benign photoresponsive groups adapted for agricultural matrices.

In recent designs, Feng et al. explored photo-initiated polymerization techniques using starch backbones as substrates to form hydrogels with both pH and light-responsive behavior. These systems incorporated poly(N-isopropylacrylamide) and polyacrylamide as co-networks for temperature and light responsiveness, enhancing release modulation in multilayer CRFs [54].

It is important to highlight that while these systems show enhanced responsiveness under lab conditions, field-scale implementation remains a challenge. Light intensity varies with crop canopy, time of day, and soil cover, all of which can affect activation efficiency. Therefore, current strategies seek to maximize surface exposure (e.g., seed-coating applications or topsoil-layer fertilizers) to capitalize on the light-triggered mechanism effectively.

Starch derivatives, especially those modified with hydrophobic moieties or integrated with conductive agents (e.g., PDA, carbon-based nanomaterials), have further expanded the material choices for light-triggered release [85]. These enhancements not only support photothermal conversion but also stabilize the release profile under fluctuating environmental conditions.

#### 3.3.7. Redox-Responsive Mechanisms

Redox-responsive systems are engineered to release encapsulated nutrients in response to the redox conditions present in soil or plant tissues, particularly the concentration of endogenous reductants such as glutathione (GSH). These systems rely heavily on redox-labile linkages, such as disulfide bonds or thiol-responsive crosslinkers, that undergo cleavage in reductive environments, leading to polymer degradation and cargo release [65].

In the context of starch-based CRFs, redox-responsive behavior is generally achieved by incorporating redox-sensitive moieties into the starch polymer backbone or coating matrix. The unique ability of these systems to target intracellular redox environments or rhizospheric redox fluctuations ensures site-specific and temporal nutrient release aligned with crop needs [68].

Hou et al. synthesized a cellulose-based nanogel with both pH- and redox-responsiveness using 3,3′-dithiobis(propionohydrazide) as the crosslinker. While the backbone was carboxymethyl cellulose, similar strategies can be adapted for starch derivatives, particularly through the introduction of thiolated linkers or cystamine-type disulfide bridges. Upon exposure to GSH-rich zones in plant roots, the disulfide bridges cleave, leading to gel disintegration and subsequent nutrient release [86,87]. Kilic-Boz et al. demonstrated that redox-sensitive hydrogels exhibited on-demand release of large biomacromolecules like BSA under reducing conditions using N,N′-bis(acryloyl)cystamine crosslinkers. This mechanism is directly applicable to starch matrices where similar crosslinking chemistry could enable release in reductive microenvironments, such as those near root exudates [88].

From a molecular standpoint, the starch structure offers several reactive hydroxyl groups, which can be chemically modified to attach disulfide-containing linkers. These linkers undergo cleavage upon interaction with GSH or other reducing agents, disrupting the matrix and enhancing permeability. Additionally, redox-sensitive hydrogels can experience sol–gel transitions or network loosening under reduction, facilitating a burst or sustained nutrient release depending on the design [89]. The redox potential in plant rhizospheres varies with microbial activity, root respiration, and plant type. Therefore, the release from redox-sensitive CRFs is inherently dynamic and responsive to biological demand, distinguishing them from passive diffusion-controlled systems. This adaptive behavior enhances nutrient use efficiency and minimizes leaching losses [90].

In recent reviews, such as by Mansouri et al., redox-responsive materials have been recognized as emerging candidates for smart fertilizer coatings, emphasizing their compatibility with sustainable agricultural systems [89]. Moreover, starch-based redox-responsive hydrogels offer environmental advantages. Polymers derived from renewable resources, combined with biodegradable crosslinkers, ensure minimal ecological burden. Redox degradation products are typically benign and integrate into the soil organic matrix [91].

An innovative approach involves dual-responsive behavior, as shown by Hou et al., where pH and redox responsiveness co-exist, mimicking the multi-factorial cues in real soil systems. Such hybrid stimuli-responsive CRFs can be particularly advantageous in heterogeneous agricultural environments [86]. Redox-responsive starch-based coatings represent a promising frontier in CRF development. By leveraging soil redox dynamics, especially GSH gradients near roots, these systems offer spatially and temporally regulated nutrient delivery. Future work should focus on the fine-tuning of crosslinking density, degradability under field-relevant redox conditions, and interaction with soil microbiota to ensure optimal performance [92].

#### 3.3.8. Toward Climate-Smart and Precision Agriculture

These smart release behaviors align perfectly with climate-resilient farming practices. By tuning nutrient delivery in response to natural environmental cues, such as soil moisture, salinity, pH, or temperature, starch-based smart CRFs reduce fertilizer runoff, minimize environmental impact, and improve yield efficiency. The renewable nature of starch further enhances the sustainability profile of these systems, supporting low-carbon agricultural practices [93].

**Figure 5:**
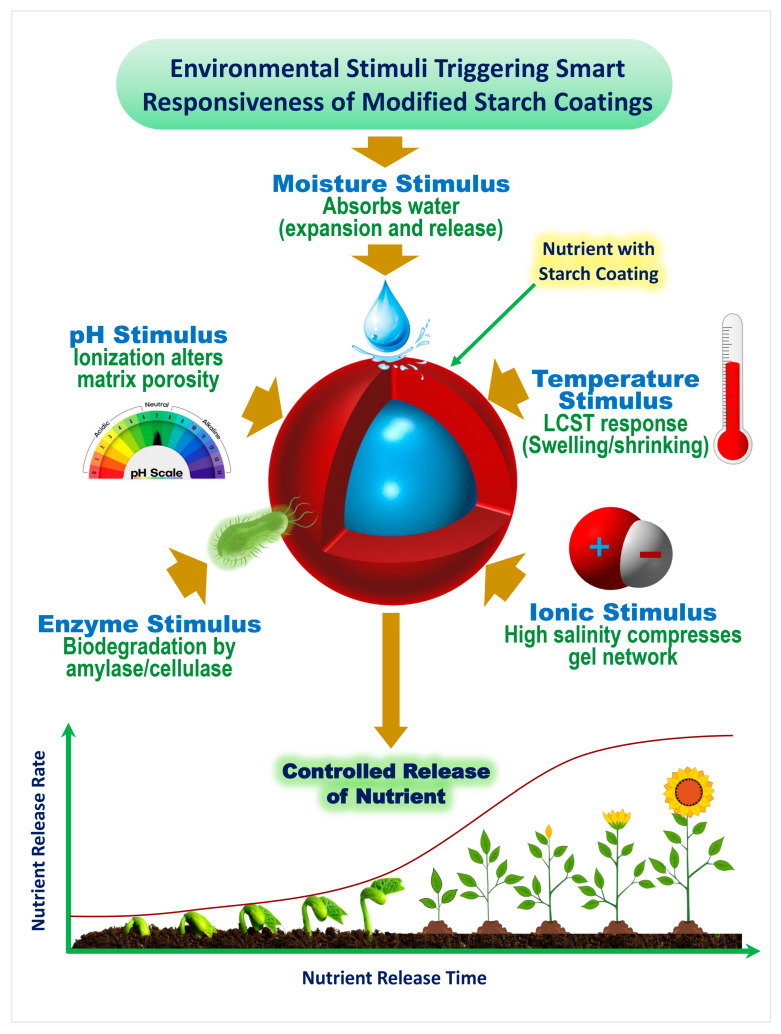
Environmental stimuli and smart response mechanisms in modified starch-based coatings for CRFs.

External triggers such as pH, temperature, moisture, enzymatic activity, and ionic strength induce reversible structural changes in the starch matrix, including swelling, shrinking, or enzymatic degradation [94]. These changes regulate the timing and rate of nutrient release, supporting climate-smart and precision agriculture practices (Figure 5).

A comprehensive summary of selected studies on starch-based CRF systems is provided in Table 1. These include various material formulations, environmental triggers, structural modifications, and nutrient-release behaviors evaluated under lab and field conditions. This compilation highlights the diversity and progress of starch-based smart fertilizers, offering comparative insight into their performance, responsiveness, and application relevance.

## 4. Fabrication and Processing Techniques

### 4.1. Solution Casting and Drying

Solution casting is a widely used technique in the fabrication of starch-based films and coatings for CRFs. This method involves dissolving or dispersing starch and additives in water or aqueous blends, followed by casting into molds and drying under controlled conditions. The simplicity, scalability, and compatibility of this method with biopolymers make it particularly attractive for developing sustainable CRF coatings [91].

A typical process begins with the gelatinization of starch under heating (typically 80–95 °C), followed by the incorporation of plasticizers such as glycerol or sorbitol. Additional components like polyvinyl alcohol (PVA) may be added to improve film strength and water resistance. The solution is poured into a mold (e.g., glass or Teflon tray) and dried at room or elevated temperature (50–80 °C), resulting in a flexible, continuous film [92].

#### 4.1.1. Film Uniformity and Mechanical Integrity

The quality of the film is largely influenced by the drying temperature, ambient humidity, and casting thickness. Rapid drying can lead to surface defects or phase separation, whereas gradual solvent evaporation promotes homogeneity. In a study combining starch and PVA, formaldehyde was used as a crosslinker to enhance tensile strength and reduce water solubility, key traits for CRF application [91]. Blending starch with biodegradable polymers such as PBAT (polybutylene adipate terephthalate) and casting at moderate temperatures resulted in densified films with better water resistance and mechanical durability [93].

#### 4.1.2. Role of Additives

Plasticizers reduce film brittleness but also influence water permeability and swelling. For example, films plasticized with glycerol demonstrated higher elongation at break but also faster nutrient release due to increased hydrophilicity. On the other hand, the addition of cellulose nanofibers or silica can modulate film porosity and release behavior. Notably, starch–PVA films cast with crosslinkers displayed superior barrier properties against NH_4_^+^ migration, which is critical for regulating nutrient leaching [91,92].

#### 4.1.3. Drying and Controlled-Release Behavior

The drying process plays a crucial role in establishing the internal structure of the coating. Higher drying temperatures yield denser networks, which slow down nutrient diffusion. In one study, films cast and dried from a starch–PBAT matrix demonstrated significantly lower water uptake and slower urea release under simulated rainfall, making them suitable for semi-arid agricultural conditions [93].

#### 4.1.4. Hydrogel Formation via Controlled Casting

Under appropriate casting and drying conditions, solution-cast films can retain hydrogel-like behavior, particularly if they are not fully dehydrated. This semi-interpenetrating network can offer swelling-triggered nutrient release. For instance, a starch hydrogel system formed via solution casting showed prolonged moisture retention and steady release over several days in soil incubation trials [94].

#### 4.1.5. Critical Process Parameters

- Drying Rate: should be optimized to avoid skin formation or brittleness.
- Crosslinking Level: controls film strength and dissolution rate.
- Polymer Blends: influence flexibility, water uptake, and biodegradability.
- Thickness Control: directly affects nutrient diffusion rate.

Solution casting remains a core technique for fabricating starch-based CRF films. Its versatility allows fine-tuning of structural, mechanical, and release properties, making it suitable for both lab-scale studies and pilot-scale production of smart agrochemical delivery systems [95].

### 4.2. Fluidized-Bed Spray Coating (Wurster, Top-Spray, Bottom-Spray)

Fluidized-bed spray coating is one of the most scalable and controllable fabrication techniques used for developing CRFs, especially when using starch-based and biodegradable polymers. This technique ensures uniform coating and allows for fine-tuning of coating parameters such as droplet size, spray rate, atomization pressure, and fluidizing air temperature to control the microstructure and release behavior of coated granules [117].

In this process, fertilizer granules are fluidized in a vertical column using a stream of air, while a polymer solution is sprayed from a nozzle. As droplets impinge and spread over the particles, a film is formed and subsequently dried by the heated air. The result is a uniform coating that can be modulated in thickness, porosity, and mechanical strength [117].

Recent studies have emphasized the effectiveness of this technique for coating urea granules using ethyl cellulose, starch–PVA blends, and even lignocellulosic materials. For instance, one work employed a response surface methodology to optimize spray rate, atomization pressure, and solution concentration to achieve desirable coating thickness and nutrient release duration [118].

Starch and starch derivatives, due to their hydrophilicity and biodegradability, are typically blended with hydrophobic agents like PVA, citric acid, or waxes to form stable, semi-permeable films. When applied via fluidized bed, these blends result in reduced burst release, improved film adhesion, and minimized surface abrasion [92].

Wurster coating, a modified bottom-spray technique, is particularly favored for starch-based CRFs as it enhances coating uniformity and reduces agglomeration. In a recent example, starch was modified with citric acid and coated on urea in a Wurster-type coater, which resulted in a prolonged release over 28 days and excellent resistance to impact damage [119].

Comparatively, this technique offers several advantages over pan and rotary coaters: better granule suspension, more consistent film build-up, and shorter drying time. Furthermore, by using biopolymer composites with nanofillers or plasticizers, researchers have successfully developed stimuli-responsive coatings with water vapor permeability tailored for environmental triggers such as humidity and temperature [79].

In another investigation, granules coated with lignin–starch blends showed a dual-mode release pattern: an initial slow diffusion-controlled phase followed by polymer degradation. This dual mechanism is effectively leveraged in fluidized bed systems where in situ film curing and post-treatment annealing can be applied [76].

Given the rapid advancement in CRF development and sustainability goals, the integration of biodegradable starch-based polymers with optimized fluidized-bed processing is expected to dominate future commercial fertilizer technologies.

### 4.3. Crosslinking and Chemical Grafting Routes

Chemical crosslinking and grafting are essential strategies to tailor the structural and functional properties of starch-based coatings in CRFs. These approaches not only enhance the mechanical strength, water resistance, and structural stability of the coating matrix but also provide precise control over the nutrient release kinetics by tuning the network density and hydrophilicity of the system.

Crosslinking involves forming covalent or ionic bonds between starch chains and crosslinkers, creating a three-dimensional polymeric network. Common crosslinking agents include borax, citric acid, epichlorohydrin, glutaraldehyde, and sodium trimetaphosphate (STMP). For instance, borax-mediated ionic crosslinking enhances the self-healing ability and structural integrity of acrylamide-grafted starch hydrogels, allowing them to withstand repeated swelling–de-swelling cycles while maintaining slow nutrient release characteristics [120].

Citric acid and STMP, being biodegradable and non-toxic, have also been widely used as green crosslinkers. These agents not only reinforce the hydrogel matrix but also regulate the degradation profile of the coating, allowing for synchronization between fertilizer release and crop nutrient demand [96]. Crosslinking also significantly impacts water retention and diffusion pathways by modulating the porosity and crystallinity of the starch network, which ultimately governs nutrient migration rates.

Chemical grafting, on the other hand, involves the attachment of synthetic polymer chains onto the native starch backbone through radical-initiated polymerization. This method is especially useful for introducing functional groups (e.g., –COOH, –NH_2_) that enhance the responsiveness of starch-based materials to environmental triggers such as pH and temperature. Acrylic acid, acrylamide, and their copolymers are commonly grafted onto starch using redox initiators like ceric ammonium nitrate (CAN) or potassium persulfate (KPS). These grafted networks exhibit high swelling capacity, tunable hydrophilicity, and improved encapsulation efficiency [121].

The grafting ratio and degree of crosslinking play a synergistic role in determining the nutrient release behavior. A higher crosslink density generally leads to slower water penetration and delayed nutrient diffusion, whereas optimal grafting ensures the desired responsiveness and structural flexibility. Moreover, chemical grafting also allows for the formation of interpenetrating polymer networks (IPNs) when blended with other biopolymers or synthetic polymers, further broadening the functional scope of starch-based CRFs [122].

Mansouri et al. emphasized that both crosslinking and grafting approaches are not mutually exclusive but can be effectively combined to design high-performance CRF coatings. For example, grafted starch networks further reinforced via crosslinking demonstrate superior barrier properties, reduced burst release, and improved mechanical stability under soil stress conditions [79].

The chemical modification of starch via crosslinking and grafting is a powerful tool to engineer intelligent delivery systems that align with sustainable agricultural practices. By adjusting the type and concentration of crosslinkers and grafted monomers, researchers can fine-tune the release kinetics, environmental responsiveness, and mechanical integrity of CRF coatings tailored for diverse field applications.

### 4.4. Encapsulation and Hydrogel Bead Formation

Encapsulation of nutrients within hydrogel beads has emerged as a promising strategy for enhancing the efficiency of CRFs. This technique leverages the water-absorbing capacity and structural tunability of hydrogels to encapsulate fertilizers and modulate their release under different environmental conditions. Hydrogel beads act as both physical barriers and nutrient reservoirs, enabling sustained release while protecting encapsulated materials from premature dissolution and environmental losses.

Alginate-based hydrogels are among the most commonly employed systems for encapsulation due to their biocompatibility, biodegradability, and mild gelation conditions. For instance, calcium ion-induced crosslinking of sodium alginate forms hydrogel networks suitable for encapsulating nutrient solutions like urea, potassium nitrate, or struvite [61]. In a recent study, urea was intercalated into montmorillonite layers and then encapsulated using alginate gelation, resulting in beads that exhibited enhanced nutrient retention and slow-release behavior due to dual entrapment, within the clay matrix and the alginate shell [123].

Another innovation involves the use of composite beads integrating natural polymers like κ-carrageenan with starch or chitosan. These biopolymer matrices support hydrogel formation under gentle conditions and allow fine-tuning of release properties via polymer concentration, ionic crosslinkers, and drying methods. For example, κ-carrageenan hydrogels crosslinked with potassium ions successfully encapsulated urea and demonstrated pH- and temperature-responsive release behavior, particularly advantageous for field conditions where environmental fluctuations are common [124].

Incorporation of humic acid or poly(acrylic acid) into hydrogel matrices has also been shown to enhance performance. In one study, hydrogel beads made from sodium alginate and poly(acrylic acid), with or without humic acid, were used to encapsulate struvite, a slow-release source of nitrogen, phosphorus, and magnesium. These beads improved nutrient uptake and reduced sodium stress in saline soils, showcasing both agronomic and environmental benefits [125].

The structural and functional optimization of hydrogel beads extends beyond polymer selection. Strategies such as the addition of layered double hydroxides, nanoclays, or mineral carriers have been explored to boost mechanical stability and loading efficiency. Additionally, encapsulation within beads offers an excellent opportunity for co-delivery of nutrients and soil conditioners, like biochar or zeolites, creating multifunctional delivery systems tailored to site-specific needs [126].

Despite promising results at the laboratory scale, scaling up the encapsulation process and ensuring field durability under varying agro-climatic conditions remain challenges. Future research should focus on improving bead mechanical integrity, controlling swelling-induced disintegration, and ensuring cost-effective production routes compatible with industrial fertilizer manufacturing.

### 4.5. Use of Nanomaterials and Reinforcing Additives

Incorporating nanomaterials such as clays, silica, nanozeolites, and carbon-based nanostructures into hydrogel matrices has emerged as a promising strategy to enhance the mechanical, thermal, and functional performance of CRFs. These nanofillers provide reinforcement by forming hydrogen bonds or electrostatic interactions with the polymer matrix, resulting in improved structural integrity, swelling control, and prolonged nutrient release.

Montmorillonite (MMT) clay, a naturally occurring layered silicate, has been extensively used as a reinforcing additive in hydrogel matrices. A recent study by Jumpapaeng et al. (2023) developed biodegradable nanocomposite hydrogels based on cassava starch (Cst), natural rubber (NR), and polyacrylamide (PAM), incorporating various MMT contents. These nanocomposite hydrogels exhibited improved tensile strength, water retention capacity, and biodegradability. Notably, a formulation with 3 wt% MMT achieved a swelling ratio of 7074% and enhanced nitrogen release and mechanical performance compared to the MMT-free system [97].

Silica-based nanomaterials, including nano-silica (SiO_2_), have also been used to enhance coating strength and moisture resistance. In a study by Zhao et al. (2022), urea was coated with a biochar–nano-silica hybrid layer, resulting in improved nutrient retention and reduced release rate in soil columns, especially under high-moisture conditions. The presence of silica contributed to surface roughness and controlled porosity, which acted as a diffusion barrier for nitrogen [127].

Cellulose nanofibers (CNFs), another bio-based reinforcing filler, provide a renewable and biodegradable option for improving film strength and reducing water sensitivity. Nasri-Nasrabadi et al. (2014) prepared thermoplastic starch composites reinforced with CNFs derived from rice straw. Incorporation of 10 wt% CNFs enhanced Young’s modulus, yield strength, and humidity absorption resistance, although a decrease in transparency was noted [98].

Nanozeolites, with their high surface area and cation-exchange capacity, have been explored for their ability to bind ammonium and other nutrient ions. Studies show that nanozeolite–chitosan composites improve nutrient retention while also offering controlled swelling and biodegradability, as demonstrated in composite matrices for CRFs [99].

Other nanofillers, such as graphene oxide (GO) and cellulose nanocrystals, have shown potential due to their unique barrier properties and high aspect ratios. For example, GO–starch composites displayed enhanced encapsulation of nutrients and prolonged release profiles in soil-based tests [100].

The combination of these nanomaterials with starch or other biopolymers enables the fabrication of CRF coatings that not only meet agronomic requirements but also contribute to sustainability goals by reducing synthetic polymer usage and minimizing environmental leaching.

### 4.6. Layer-by-Layer Assembly and Multilayer Coatings

Layer-by-layer (LbL) assembly and multilayer coating techniques have emerged as powerful approaches for fabricating CRF coatings with precise structural tunability, mechanical robustness, and stimuli-responsive behavior. This method involves the alternate deposition of oppositely charged materials, typically polyelectrolytes, forming nanostructured films with controllable thickness and composition.

Recent studies have explored the potential of polyelectrolyte multilayer (PEM) films to regulate nutrient release kinetics by adjusting layer numbers, charge densities, and ionic crosslinking. These multilayers create physical diffusion barriers and can respond to environmental cues such as pH, temperature, or ionic strength, enabling programmable nutrient delivery [128]. For example, bio-based polyelectrolytes like chitosan, alginate, and tannic acid have been explored as environmentally friendly building blocks for LbL films [129].

In a recent development, programmable bionanocomposite coatings using polyurethanes and exfoliated nanoclays were fabricated via LbL-like processes, yielding slow-release nitrogen fertilizers with controlled porosity and strong structural integrity [130]. These films not only improved nutrient use efficiency (NUE) but also demonstrated a reduction in nitrogen leaching and volatilization under field conditions.

Multilayer coatings also offer the advantage of incorporating functional additives at different layers. For instance, outer layers can include water-repellent waxes or anti-burst polymers, while inner layers focus on nutrient encapsulation and protection. This hierarchical design enables dual or multiple release phases, essential for matching crop nutrient demands throughout different growth stages [131].

Additionally, layer thickness and crosslinking density critically affect nutrient permeability. Denser multilayers or those formed with divalent ion crosslinking (e.g., Ca^2+^) significantly prolong the release profile of urea and other nutrients [132]. The integration of bio-nanofillers like bentonite, nano-silica, or cellulose nanocrystals in specific layers further enhances film durability and controls swelling behavior [130].

**Figure 6:**
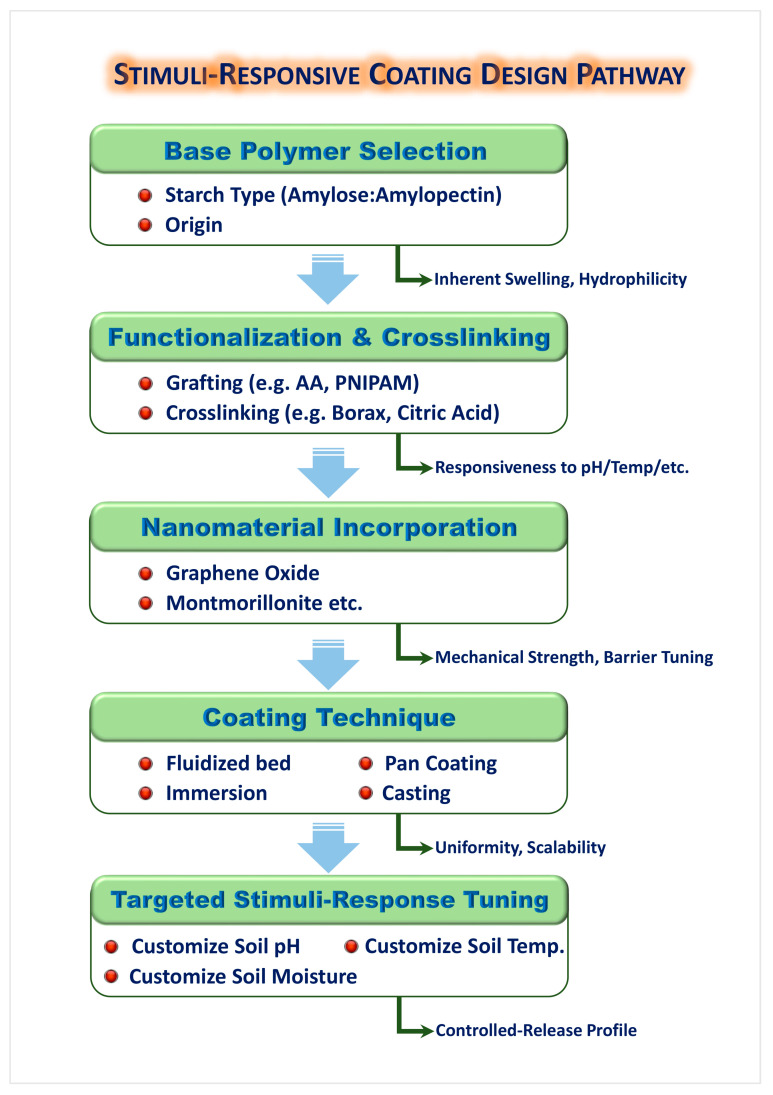
Stepwise design pathway for developing stimuli-responsive starch-based coatings for CRFs.

Overall, LbL and multilayer coatings represent a promising frontier in sustainable CRF development. Their modular design, adaptability to various feedstocks, and compatibility with stimuli-responsive features make them suitable for next-generation fertilizers targeting resource efficiency and environmental safety. A comprehensive design framework for developing stimuli-responsive starch-based coatings is illustrated in Figure 6.

## 5. Evaluation and Characterization of Starch-Based Stimuli-Responsive CRFs

### 5.1. Morphological, Thermal, and Mechanical Analysis

The structural characterization of starch-based CRFs is essential to assess the effectiveness and reliability of the coating system under agricultural conditions. These evaluations are typically performed using morphological imaging, thermal stability assessments, and mechanical integrity tests, which together provide a holistic understanding of the material’s performance [60].

#### 5.1.1. Morphological Analysis

Morphological evaluation, typically performed through scanning electron microscopy (SEM), reveals the surface smoothness, porosity, crack formation, and coating uniformity of the fertilizer granules [68]. A uniform and compact surface morphology is indicative of enhanced barrier properties that can effectively slow nutrient release. For example, starch–PVA-based hydrogel-coated fertilizers demonstrated a relatively smooth and dense surface in SEM images, suggesting efficient encapsulation and minimal coating defects. The inclusion of fillers such as biochar or nano-SiO_2_ has been shown to further improve the microstructure by reducing micropores and forming tighter networks [90,127].

#### 5.1.2. Thermal Analysis

Thermal Gravimetric Analysis (TGA) and Differential Scanning Calorimetry (DSC) are commonly used to investigate the thermal stability of biopolymer coatings. Starch-based composites typically exhibit two-stage weight loss: initial water evaporation followed by polymer decomposition. Incorporation of nano-additives or crosslinking agents raises the decomposition temperature, indicating improved thermal resistance. In one study, starch hydrogels with sodium alginate and polydopamine showed significantly delayed degradation onset, confirming thermal reinforcement through molecular interactions [63].

#### 5.1.3. Mechanical Properties

The mechanical strength of coating materials ensures their survival during transportation and handling. Tests such as tensile strength and compressive resistance help evaluate robustness. Films containing plasticizers like glycerol often show improved flexibility but reduced tensile strength. Conversely, reinforcement with activated carbon or nano-silica markedly enhances tensile properties by increasing intermolecular hydrogen bonding and reducing polymer chain mobility. Moreover, swelling behavior tests provide indirect insights into coating integrity under humid conditions; lower swelling ratios often correlate with mechanically stable networks [52].

Together, these analyses highlight the critical role of composite formulation in tuning the physical stability and nutrient release behavior of starch-based CRFs. A tailored combination of structural modifiers and crosslinking strategies ensures that the coatings maintain their functional properties over time, even under fluctuating environmental conditions.

### 5.2. Swelling Ratio, Biodegradability, and Water Retention

The swelling behavior of hydrogels plays a pivotal role in determining their nutrient release kinetics and water retention capabilities. Swelling capacity is governed by multiple factors, including the nature of the polymeric backbone, degree of crosslinking, and environmental conditions such as temperature, pH, and ionic strength. In starch-based hydrogel systems, the hydroxyl-rich structure facilitates high water absorption through hydrogen bonding, which leads to substantial swelling. Pellá et al. reported that activated carbon incorporation led to enhanced swelling performance due to its porous structure and water affinity [70]. Similarly, a maximum swelling ratio of ~170% was observed, which was attributed to the synergistic interactions between the hydrophilic matrix and embedded dopamine units [63].

The swelling behavior of hydrogel-based CRFs is not only essential for nutrient diffusion but also dictates water-holding capacity and physical stability in the soil. Quantitative swelling ratios under different pH or ionic conditions are frequently employed to simulate real-world agricultural environments [133]. For instance, starch–alginate hydrogels showed pH-sensitive swelling from 520% at pH 4.0 to over 680% at pH 7.0, attributed to enhanced electrostatic repulsion between carboxylate ions in the network under neutral conditions. Similarly, salt-tolerant swelling behavior was validated with simulated saline water, where hydrogels retained 50–60% of their maximum swelling even in 0.9 wt% NaCl solution, indicating robustness under irrigation with marginal water [134].

Water retention is another critical parameter for evaluating the agronomic viability of hydrogel-based CRFs. Hydrogels with high water retention capacity can reduce irrigation frequency and improve nutrient utilization efficiency. According to Pellá et al. [70], the developed formulations retained more than 50% of their absorbed water over 7 days under ambient conditions, significantly outperforming unmodified starch gels. The inclusion of crosslinked PVA and nano-additives was found to create a more tortuous network that reduced evaporative losses. In terms of water retention, hydrogel-treated soils consistently showed prolonged water-holding capacity. For instance, soil with hydrogel amendments retained moisture up to 9 days longer than untreated soil under identical conditions. This moisture extension was particularly noticeable in sandy soils where capillary forces are weak.

Regarding biodegradability, starch-based hydrogels offer an eco-friendly advantage due to their natural origin and susceptibility to microbial degradation. Cano et al. [90] revealed that pure starch–PVA films exhibited more than 60% weight loss within 30 days of soil burial, indicating robust biodegradability. However, the presence of certain antimicrobial agents like citric acid slowed down microbial colonization and enzymatic degradation, highlighting a potential trade-off between preservation and biodegradation. This factor must be carefully considered during hydrogel formulation to ensure environmentally safe disintegration post application. Starch-based composites were shown to lose approximately 30–35% of their mass over a 30-day period under composting conditions. For example, starch–PVA blends with biochar and nano-SiO_2_ additives degraded more slowly, suggesting their persistence was enhanced due to filler–matrix interactions. Additionally, mass loss studies under soil burial tests revealed that enzyme activity significantly affects the degradation rate of starch–polymer composites, especially when natural polysaccharides dominate the matrix [135].

Collectively, the swelling ratio, water retention efficiency, and biodegradability profile underscore the multifunctional performance of starch-based hydrogel CRFs and their promise for sustainable agricultural applications.

### 5.3. Release Kinetics Under Controlled Stimuli

Evaluating the nutrient release kinetics of stimuli-responsive starch-based hydrogels is essential to understanding their performance and optimizing their formulation. Various mathematical models have been employed to describe nutrient diffusion and release behavior under environmental triggers like moisture, pH, or temperature [136].

The zero-order model [137] assumes a constant release rate, independent of concentration, and is expressed as

(1) $$M_{t}=k_{0}t$$

where *M_t_* is the amount of nutrient released at time *t*, and *k*_0_ is the zero-order release constant. This model is ideal for systems that maintain a steady nutrient supply, particularly in hydrogels designed for uniform release profiles.

The first-order model, commonly used for systems where the release rate is concentration-dependent, is given by [138]

(2) $$\ln\left(1-\frac{M_{t}}{M_{\infty }}\right)=-k_{1}t$$

Here, *M*_∞_ represents the total nutrient available for release, and *k*_1_ is the first-order kinetic constant.

The Higuchi model [139], derived for diffusion-controlled release systems, follows the equation below:

(3) $$M_{t}=k_{H}\sqrt{t}$$

where *k*_*H*_ is the Higuchi dissolution constant. This model applies well to matrix-based hydrogels where nutrient diffusion is governed by Fickian mechanisms.

A more general model is the Korsmeyer–Peppas model [140], suitable for distinguishing between Fickian and non-Fickian transport mechanisms:

(4) $$\frac{M_{t}}{M_{\infty }}=k_{KP}t^{n}$$

In this case, *k*_*KP*_ is the kinetic rate constant, and *n* is the release exponent. The value of *n* provides insight into the mechanism; Fickian diffusion (*n* ≤ 0.5), anomalous transport (0.5 < *n* < 1), or Case II transport (*n* = 1).

The Hixson–Crowell model [141], which accounts for changes in the surface area and diameter of particles during release, is represented by

(5) $$M_{0}^{\frac{1}{3}}-M_{t}^{\frac{1}{3}}=k_{HC}t$$

Here, *M*_0_ is the initial amount of nutrient, and *k*_*HC*_ is the Hixson–Crowell constant.

Finally, the Weibull model [142], which is highly flexible and empirical, fits a wide range of release curves and is expressed as

(6) $$\frac{M_{t}}{M_{\infty }}=1-e^{-{\left(\frac{t}{\alpha }\right)}^{\beta }}$$

where *α* and *β* are the scale and shape parameters, respectively.

The evaluation of nutrient release kinetics is fundamental to understanding the performance of stimuli-responsive hydrogels under varying environmental conditions. Starch-based CRFs respond to external triggers, such as pH, temperature, ionic strength, and moisture, by altering their network structure, which consequently governs the nutrient release profile [7]. Various kinetic models are employed to describe and predict these behaviors, including first-order, Higuchi, Korsmeyer–Peppas, and parabolic diffusion models.

In one study, alginate–calcium crosslinked starch hydrogels encapsulating phosphate fertilizers showed release behavior that followed pseudo-second-order and Higuchi models, highlighting the diffusion-dominated release mechanism in moist soil environments [52].

Similarly, a kinetic evaluation of humic acid-loaded starch-based hydrogels under varying pH conditions demonstrated a clear dependence of release rate on environmental acidity. The release was found to be faster at pH 5 compared to neutral or basic environments, owing to partial protonation of carboxylic groups within the hydrogel network. This protonation weakens ionic crosslinks formed with multivalent cations, increasing chain mobility and osmotic water uptake. The resulting network expansion enhances water penetration and diffusivity, thereby accelerating nutrient release [60].

Furthermore, stimuli-responsive starch hydrogels incorporating cellulose or activated carbon exhibit temperature- and moisture-sensitive swelling behavior. This swelling affects polymer chain mobility and subsequently regulates the nutrient diffusion rate. Modeling such systems using the Korsmeyer–Peppas equation has shown release exponent (*n*) values between 0.45 and 0.89, indicating anomalous (non-Fickian) transport mechanisms combining diffusion and polymer relaxation [143].

In another evaluation, a kinetic modeling study applied the Ritger–Peppas model to quantify release rates from thermally crosslinked hydrogels loaded with urea. These systems exhibited zero-order release in saturated moisture conditions, ideal for maintaining constant nutrient availability over prolonged durations [144].

Finally, starch-based composite systems reinforced with SiO_2_ or bentonite clay demonstrated multi-modal release kinetics, showing biphasic release: an initial burst followed by a sustained, controlled phase. This dual behavior is suitable for staged nutrient delivery aligning with plant uptake patterns [138].

**Figure 7:**
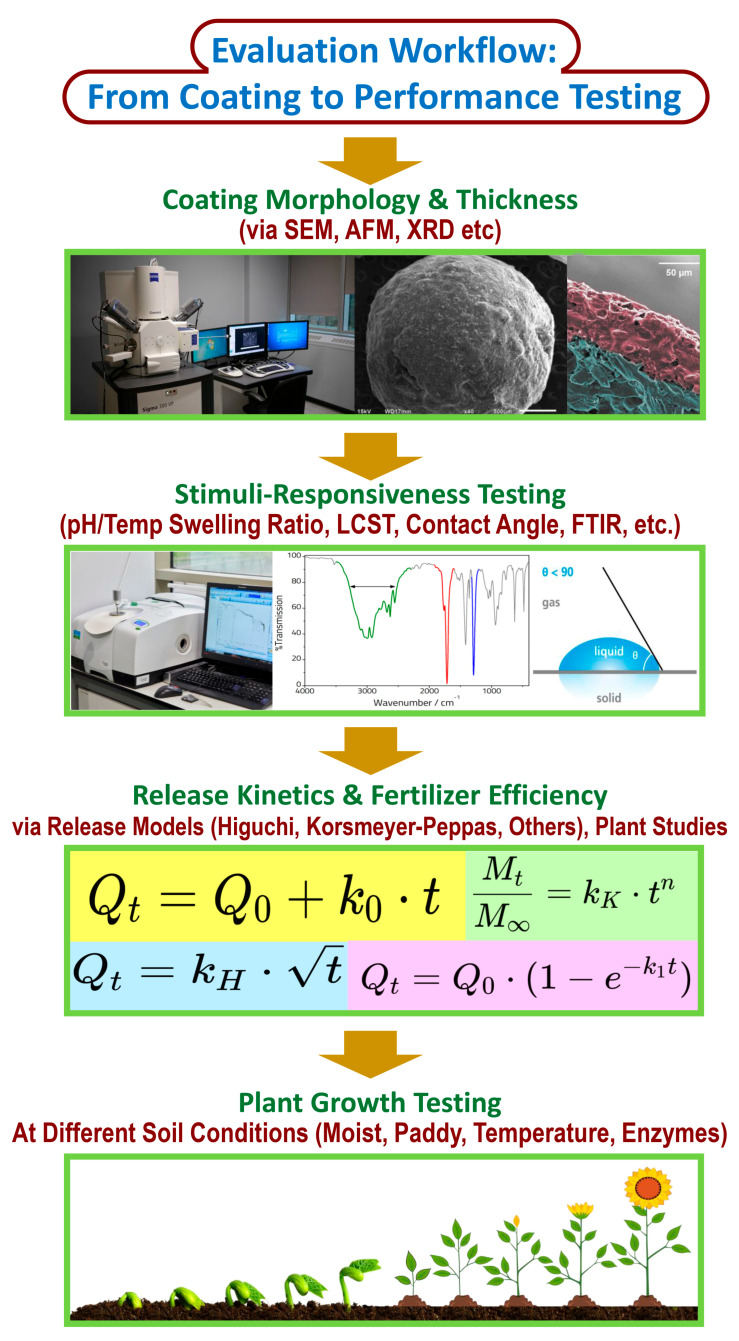
Evaluation workflow for stimuli-responsive starch-based fertilizer coatings.

Modeling and experimental data suggest that starch-based hydrogels enable tunable nutrient release under environmentally relevant stimuli. The choice of kinetic model and fitting parameters offer insight into underlying transport mechanisms, which are crucial for the rational design of next-generation CRFs. A comprehensive workflow highlighting the characterization and performance evaluation stages of stimuli-responsive starch-based fertilizer coatings is presented in Figure 7.

A summary of the key evaluation aspects, analytical methods employed, and their specific purposes is presented in Table 2.

## 6. Smart Performance of Starch-Based CRFs Under Field-Simulated Conditions

### 6.1. Performance in Various Soils

The performance of starch-based CRFs under diverse soil conditions, acidic, alkaline, and saline, is critical for their practical applicability in global agriculture. Soil pH and salinity significantly influence nutrient release behavior, plant nutrient uptake, and microbial degradation of biopolymer coatings [93].

In acidic soils, the proton-rich environment tends to accelerate the hydrolysis of biopolymeric coatings, especially those containing ester or amide linkages. Wang et al. (2023) observed that carboxymethyl starch-based coatings exhibited higher nutrient release rates in acidic conditions due to enhanced hydrolytic cleavage of glycosidic bonds, facilitating nutrient availability in low-pH soils [145]. This rapid release can be beneficial for short-cycle crops but may compromise controlled delivery over extended periods.

In alkaline soils (pH > 7.5), weak-acid groups on starch derivatives (e.g., –COOH) deprotonate to –COO^−^, increasing fixed charge density and—under low ionic strength—promoting electrostatic repulsion and counter-ion osmotic pressure that enhance swelling and water uptake, which can accelerate nutrient diffusion. However, many alkaline/calcareous soils also contain elevated salts and Ca^2+^/Mg^2+^; charge screening and divalent-cation bridging can suppress swelling and slow diffusion. Consistent with this, Qiao et al. (2016) reported that starch-g-poly(acrylic acid) coatings maintained prolonged release in alkaline environments, attributable to limited effective chain expansion under these ionic conditions [101]. These coatings are particularly suited for nutrient management in arid and calcareous soils.

Saline soils present an added challenge due to high osmotic stress and ionic strength. These conditions may restrict water uptake into the coating matrix, thus suppressing polymer swelling and delaying release. However, certain salt-tolerant biocomposite formulations, such as starch blended with humic acid or bentonite, have shown improved performance. In a field-simulated study, Motamedi et al. (2023) reported that urea encapsulated in starch–silica nanocomposites exhibited stable release profiles even in saline soils with electrical conductivity exceeding 5 dS/m [146]. The incorporation of nanofillers likely mitigated structural collapse under saline stress by reinforcing the polymer network.

Moreover, biodegradable starch coatings showed pH-sensitive release and degradation behavior across soil types. For instance, the work of Chiaregato et al. (2021) indicated that composite films based on carboxymethyl starch and polyvinyl alcohol degraded more rapidly in acidic and neutral soils, aligning with increased microbial activity and moisture retention [102].

The soil-specific response of starch-based CRFs highlights the need for adaptive formulation strategies. Incorporating hydrophobic agents for acidic soils, hydrophilic enhancers for alkaline soils, and osmo-regulatory additives for saline environments can optimize performance. This responsiveness underscores the suitability of starch-based coatings in precision agriculture and site-specific nutrient management [103,104,147].

### 6.2. Controlled Release Under Environmental Variability

The performance of starch-based CRFs is significantly influenced by environmental variables such as temperature, humidity, soil moisture, and pH. These parameters alter the physicochemical behavior of the polymeric matrix, modulating the diffusion, swelling, and degradation processes that govern nutrient release. Smart starch-based coatings have been designed to respond predictably to such stimuli, enabling climate-adaptive nutrient management and reducing environmental losses [64].

Temperature is a primary driver of polymer mobility and diffusion kinetics. With increasing temperature, the molecular motion within the starch-based matrix becomes more dynamic, accelerating water uptake and polymer relaxation, which in turn enhances nutrient release. For instance, Yang et al. (2020) observed that hydrogel-coated urea released nitrogen more rapidly at elevated temperatures due to faster swelling and higher degradation rates of the polymer shell [46]. Similarly, Xu et al. (2023) demonstrated that gelatinized starch matrices underwent more pronounced chain relaxation above 30 °C, reducing the tortuosity of diffusion pathways and facilitating nutrient escape [53].

Moisture availability also plays a crucial role in triggering hydrogel swelling. In dry conditions, the polymer matrix remains compact, limiting water penetration and slowing nutrient release. Upon rewetting, hydrophilic functional groups such as hydroxyl and carboxyl in starch derivatives rapidly attract water, enabling rapid swelling and nutrient diffusion. This “on-demand” release mechanism has been reported by Simão et al. (2024), where starch-grafted copolymers responded sharply to soil moisture fluctuations, ensuring nutrient availability aligned with plant water uptake [77].

Soil pH affects the ionization state of functional groups within the starch matrix, thereby influencing polymer solubility and ionic interactions. In acidic or alkaline soils, the hydrogen bonding and electrostatic interactions are altered, modifying the polymer conformation and permeability. Wang et al. (2021) showed that CRFs based on carboxymethyl starch released phosphorus faster in acidic soils due to increased matrix dissolution, while alkaline pH induced crosslink contraction, slowing the release [148].

Moreover, multi-stimuli responsive systems have been engineered by incorporating additives such as polydopamine, alginate, or silica nanoparticles, which enhance the sensitivity and adaptability of the coating. For example, the composite coating developed by Simão et al. (2025) exhibited dual sensitivity to moisture and temperature, showing synchronized nutrient release with fluctuating weather patterns, a feature especially valuable in arid or monsoon-prone regions [7].

The interplay between environmental variables and polymer behavior is often described using kinetic models. The release profiles typically fit first-order or Higuchi models under isothermal conditions, but non-Fickian or anomalous transport dominates under fluctuating field conditions [149]. This complexity necessitates advanced modeling and empirical calibration to predict release behavior accurately under real-world scenarios.

In essence, environmental variability represents both a challenge and an opportunity in starch-based CRFs. Smart coatings engineered with stimuli-responsive features offer a pathway to synchronize nutrient delivery with crop needs, enhancing fertilizer efficiency and environmental sustainability.

### 6.3. Agronomic Impacts: Uptake, Growth, Yield

Starch-based CRFs have demonstrated significant agronomic advantages by enhancing nutrient uptake efficiency, stimulating plant growth, and improving crop yield [1]. These improvements stem from their ability to provide a sustained nutrient supply while reducing losses through leaching, volatilization, and runoff.

In tomato plants, the application of a pH-responsive hydrogel formulation not only enhanced water use efficiency but also significantly improved shoot and root biomass, leaf area, and fruit yield compared to conventional fertilizers. The slow and consistent nutrient release better matched plant uptake patterns, optimizing growth performance [150].

Similarly, hydrogel-based fertilizers tested on Brassica napus (oilseed rape) in pot experiments led to higher nitrogen use efficiency (NUE) and improved shoot length, chlorophyll content, and overall biomass production, as compared to uncoated urea fertilizers. The hydrogel coating provided a prolonged nitrogen release period that supported sustained vegetative development [151].

Another study on wheat (Triticum aestivum) demonstrated that CRF-coated urea formulations increased grain yield by 18.6% compared to controls, attributed to enhanced root growth and prolonged nutrient availability in the rhizosphere [152].

In the context of leafy vegetables, starch-based hydrogel fertilizers applied to lettuce resulted in a higher leaf area index, increased fresh biomass, and greater nutrient accumulation. These benefits were particularly evident under water-limited conditions, highlighting the dual function of CRFs as nutrient carriers and water-retaining agents [153].

Likewise, the controlled trials of Zhu et al. showed that smart hydrogel-based fertilizers boosted plant height, nutrient use efficiency, and fresh yield. The formulation minimized salt stress by regulating ionic release, which proved beneficial in marginal soils [154].

These findings demonstrate that starch-based CRFs hold considerable potential to enhance crop performance by providing synchronized nutrient delivery, improving water retention, and ensuring a favorable rhizosphere environment that supports robust plant growth and higher yields.

### 6.4. Environmental Fate and Degradation of Coating

The environmental fate of starch-based coatings in CRFs is critical for assessing their long-term sustainability and compatibility with agroecosystems. These coatings, derived from natural biopolymers and modified with nanomaterials or synthetic additives, undergo degradation influenced by environmental variables such as microbial activity, temperature, pH, and soil moisture [130].

Starch, as a biodegradable polymer, provides a natural pathway for environmental assimilation through enzymatic hydrolysis and microbial digestion. The rate and completeness of degradation depend on its structural features and the nature of the crosslinking or grafted polymers. For instance, coatings reinforced with materials like poly(vinyl alcohol) or lignin exhibit enhanced mechanical stability but delayed biodegradation compared to native starch [105].

Studies show that environmental degradation of starch-based coatings proceeds in multiple phases: initial surface erosion, enzymatic fragmentation, and eventual microbial mineralization. In aerobic soil environments, microbial consortia accelerate breakdown via amylase enzymes that hydrolyze α-1,4 glycosidic bonds. In contrast, anaerobic conditions, commonly found in waterlogged or compacted soils, slow the degradation process, posing a potential risk of coating accumulation [155].

Nanocomposite coatings incorporating materials such as SiO_2_, lignin, or chitosan can affect the degradation pathway. For example, Zhao et al. reported that the presence of nano-SiO_2_ modulates the microbial colonization pattern and delays the onset of significant mass loss, extending the release period but raising concerns about residual persistence [127]. Similarly, the addition of graphene oxide, though enhancing barrier properties and nutrient encapsulation, leads to slower degradation in compost and soil burial studies due to reduced water permeability and microbial access [36].

Another important aspect is the mineralization and environmental assimilation of the coating breakdown products. Qiao et al. observed that starch–lignin composite films degraded into non-toxic organic acids and sugars, which could be assimilated by soil microbial flora without altering the microbial diversity or pH significantly [101]. These findings suggest that although composite coatings prolong nutrient release, they can still maintain a benign environmental profile if designed with biodegradable reinforcing agents.

Degradation assessments have also been performed under simulated environmental stress conditions. For example, field-simulated leaching columns and accelerated aging chambers demonstrate that moisture fluctuation and UV exposure are significant contributors to initiating micro-cracks and enhancing biodegradability [156]. These observations are crucial for evaluating CRF performance in arid versus humid regions.

Importantly, starch-based CRFs have shown promise in minimizing ecological risks compared to conventional synthetic polymer coatings. Recent work by Kumar et al. highlighted that such CRFs do not contribute to microplastic contamination and are easily incorporated into the carbon and nitrogen cycles after degradation [157]. Furthermore, advanced characterization methods, such as scanning electron microscopy (SEM) and Fourier transform infrared spectroscopy (FTIR), are being employed to monitor degradation morphology and chemical structure changes over time [105].

The environmental fate of starch-based coatings is influenced by their structural design, the type of reinforcements, and soil environmental conditions. Optimizing these factors can yield coatings that not only ensure efficient nutrient release but also degrade completely and safely after fulfilling their agronomic role.

## 7. Comparative Analysis and Research Gaps

### 7.1. Comparison with Other Smart Biopolymers

Smart biopolymers like chitosan, alginate, and cellulose have been extensively investigated as carriers in CRFs, each offering distinct advantages and limitations in terms of structure, functionality, and application.

Chitosan is one of the most widely studied biopolymers due to its excellent film-forming ability, biodegradability, biocompatibility, and innate antimicrobial properties. Chitosan-based CRFs have demonstrated remarkable performance in terms of prolonged nitrogen release, plant growth promotion, and enhanced biochemical attributes such as antioxidant activity and metabolite accumulation [158]. For instance, chitosan microsphere-based nitrogen fertilizers significantly improved photosynthetic efficiency, total sugar, and flavonoid content in Chinese cabbage when compared to conventional urea application. Furthermore, chitosan’s cationic nature enables strong interaction with negatively charged nutrients and soil particles, allowing better nutrient retention and reduced leaching losses [159].

Cellulose, the most abundant natural polymer on Earth, is another promising candidate for CRFs due to its excellent biodegradability and modifiability. Despite its poor intrinsic solubility and mechanical weakness, chemically modified cellulose (e.g., cellulose acetate or carboxymethyl cellulose) has been effectively employed in fertilizer encapsulation systems [160]. These cellulose derivatives can be engineered to improve nutrient retention and release properties. Membrane-forming or hydrogel-type cellulose matrices enable physically and chemically controlled nutrient release via swelling, diffusion, or bond cleavage mechanisms. Notably, cellulose-based systems may struggle with poor water retention compared to chitosan or alginate and often require crosslinking or blending to enhance performance [161].

Alginate, another popular biopolymer derived from brown seaweed, is valued for its gelling ability, low toxicity, and environmental friendliness. Alginate-based CRFs typically employ calcium ion crosslinking to form hydrogel beads or films that encapsulate nutrients. These hydrogels offer sustained nutrient release through ion exchange and gradual matrix degradation. However, their mechanical fragility and high hydrophilicity can sometimes lead to premature swelling and nutrient release, particularly in saline or alkaline environments [162].

While all three biopolymers are biodegradable and can serve as nutrient carriers, chitosan stands out for its antimicrobial action and strong nutrient binding, alginate for its gelation and swelling control, and cellulose for its structural diversity and chemical modifiability. Comparative evaluations suggest that hybrid systems combining these biopolymers could offer synergistic effects in CRF applications.

### 7.2. What Makes Starch Unique?

Among various biopolymers used for CRF applications, starch distinguishes itself through a combination of structural simplicity, functional versatility, environmental compatibility, and economic feasibility. Its unique characteristics stem from its molecular structure, widespread availability, modifiability, and tunable responsiveness to environmental stimuli [102].

#### 7.2.1. Structural and Abundance Advantages

Starch is composed primarily of amylose (linear) and amylopectin (branched), offering a dual mechanism of film formation and gelation that is not commonly observed in other natural polymers. Its availability from a range of agricultural sources (e.g., maize, potato, cassava, and rice) makes it one of the most abundant and low-cost feedstocks for sustainable material development, particularly advantageous for agricultural applications where volume and scalability are key considerations [100].

#### 7.2.2. Biodegradability and Soil Compatibility

Unlike some synthetic polymers and even certain biopolymers, starch degrades readily in the soil through enzymatic and microbial action, producing non-toxic byproducts such as glucose and CO_2_. This eliminates concerns over microplastic contamination and ensures safe integration into the soil ecosystem, supporting sustainable nutrient management practices [137].

#### 7.2.3. Ease of Functionalization

The hydroxyl groups in starch allow for a wide range of chemical modifications (e.g., crosslinking, grafting, esterification), enabling its transformation into pH-sensitive, temperature-responsive, moisture-triggered, or enzymatically degradable carriers. This makes starch especially well suited for stimuli-responsive CRFs, where nutrient release is regulated by dynamic soil and environmental conditions [44,45].

#### 7.2.4. Smart Release Behavior

Compared to other biopolymers like chitosan or cellulose, modified starch has demonstrated more predictable and tunable swelling, solubility, and permeability characteristics, particularly when used in hydrogel or film-coating systems. This allows starch-based CRFs to respond intelligently to fluctuations in soil pH, ionic strength, and moisture content, offering precision nutrient delivery aligned with plant uptake demand [51].

#### 7.2.5. Compatibility in Composite Systems

Starch readily blends with other biopolymers (e.g., chitosan, alginate) and inorganic nanoparticles (e.g., zeolites, clays, biochar) to form hybrid coatings with enhanced mechanical strength and controlled degradation. This makes it an ideal matrix in multifunctional or multilayer systems, where starch provides the biodegradable backbone while other materials contribute structural or functional reinforcement [90].

#### 7.2.6. Economic and Agro-Industrial Relevance

Given its cost-effectiveness, wide accessibility, and alignment with green chemistry principles, starch is particularly attractive in regions with agricultural biomass surplus. This supports not only fertilizer sustainability but also circular economy strategies, as starch-based materials can be derived from food/agricultural waste streams [86].

Conclusively, starch uniquely combines smart performance with biodegradability, affordability, and modifiability, positioning it as a frontrunner among sustainable polymeric materials for smart fertilizer coatings. Its ability to bridge performance with scalability is what sets it apart in the context of both laboratory innovation and field-level implementation [24].

To provide a clear side-by-side comparison for readers, Table 3 summarizes the key characteristics of starch and other biopolymers commonly used in controlled-release fertilizer coatings, including their source, biodegradability, cost, mechanical properties, functional groups, and stimuli-responsiveness.

### 7.3. Potential in Multilayer/Composite Systems

The design of multilayer and composite coating systems represents a promising frontier in the development of starch-based CRFs. These systems integrate the biodegradability and modifiability of starch with the functional reinforcement of other polymers or fillers, enabling tailored nutrient delivery profiles, enhanced barrier properties, and mechanical stability under diverse soil environments [132].

#### 7.3.1. Limitations of Single-Component Coatings

While starch alone offers a biodegradable and eco-friendly matrix, its inherent hydrophilicity and relatively low mechanical strength can result in premature swelling or rupture under wet soil conditions. These shortcomings have spurred interest in multilayer architectures, where starch forms one component of a more robust system [24].

#### 7.3.2. Advantages of Multilayer and Hybrid Configurations

Multilayer coatings allow distinct layers to serve specific functions, such as nutrient diffusion control, environmental responsiveness, and physical protection. For example, an inner layer of starch modified for pH or moisture sensitivity can enable smart nutrient release, while an outer hydrophobic or nanoparticle-reinforced shell can delay initial swelling and protect against abrasion [5].

In composite systems, starch is often blended or reinforced with chitosan or alginate, to introduce antimicrobial or gel-forming properties; cellulose derivatives, to increase tensile strength and reduce water solubility; and nanoclays, zeolites, or biochar, to modulate porosity, ion exchange, and thermal resistance. These combinations synergistically improve the structural integrity of the coating, reduce nutrient leaching, and offer more consistent release profiles [129].

#### 7.3.3. Controlled Layer Deposition Techniques

Techniques such as layer-by-layer assembly, Wurster fluidized-bed spray coating, and sequential dipping are now being employed to create precise multilayer structures. These methods allow controlled deposition of functional layers, each designed to degrade under specific environmental cues (e.g., pH, microbial activity, temperature shifts). making the release behavior increasingly programmable [7].

#### 7.3.4. Application in Harsh Soil Conditions

Composite coatings have shown superior performance under acidic, alkaline, or saline soil environments, where a single-component starch layer may degrade too rapidly. By engineering the outermost layer to buffer or shield the inner starch matrix, the integrity of the CRF can be preserved until environmental triggers initiate release [72].

#### 7.3.5. Future Opportunities in Multilayer/Composite Systems

There is growing interest in developing bio-inspired or biomimetic multilayer coatings, such as nacre-like lamellar systems or gradient-release capsules. For starch-based materials, this could mean alternating starch layers with hydrophobic biopolymers or enzyme-degradable films, allowing fine-tuned spatial and temporal nutrient delivery [156]. Furthermore, the incorporation of nanomaterials or smart sensors in these coatings could enable real-time responsiveness to soil signals, aligning fertilizer behavior with the principles of precision agriculture.

In a nutshell, multilayer and composite systems extend the functional reach of starch beyond its intrinsic limitations, transforming it into a versatile carrier platform capable of delivering nutrients intelligently and sustainably [132]. These innovations not only enhance CRF performance but also open new avenues for customizing coatings based on specific crop, soil, and climatic requirements.

## 8. Future Perspectives

Despite significant progress in the development of starch-based stimuli-responsive coatings for CRFs, several research gaps remain that limit their broader implementation and field-level success. These gaps present strategic opportunities for future research and innovation.

### 8.1. Scalability and Industrial Translation

Most starch-based CRFs are currently synthesized and tested at the laboratory scale, with limited reports on pilot or industrial-scale production. Key challenges include process uniformity, cost-effectiveness, and consistency of coating thickness and integrity during large-batch manufacturing. Methods such as fluidized bed coating and extrusion-based encapsulation need to be optimized for starch matrices, particularly when integrating multiple functional layers or nanomaterials. Collaborative efforts between academia and fertilizer manufacturers are essential to transition these materials from bench to field.

### 8.2. Multi-Stimuli Responsiveness and Customization

While progress has been made in developing starch-based coatings responsive to individual stimuli (e.g., pH, moisture, temperature), real-world soil environments present complex and overlapping stimuli. Future coatings should be engineered to respond to multiple environmental cues simultaneously, such as a combination of soil salinity, enzyme activity, and diurnal temperature cycles. This necessitates precise molecular tailoring of starch via grafting, crosslinking, or blending with smart co-polymers to achieve controlled and synergistic responsiveness.

### 8.3. Long-Term Field Validation

Much of the performance data for starch-based CRFs comes from short-term laboratory or pot trials. Longitudinal field studies across varied agro-climatic zones are lacking. There is a need to understand nutrient release kinetics, environmental degradation, and crop uptake dynamics under real agricultural conditions. Studies should also consider seasonal variations, irrigation regimes, and soil microbiome interactions, which greatly affect fertilizer efficiency and sustainability outcomes.

### 8.4. Environmental Impact and Biodegradation Profiling

Although starch is biodegradable, the breakdown products of modified starch or its composites (e.g., starch–PVA, starch–chitosan, starch–nanoparticle hybrids) require thorough environmental assessments. Research must focus on the biodegradation pathways, ecotoxicity profiles, and potential microplastic formation (if synthetic additives are used). Lifecycle assessment (LCA) tools should be employed to evaluate the carbon footprint, water usage, and overall sustainability benefits compared to conventional fertilizers.

### 8.5. Integration with Precision Agriculture Technologies

Future smart coatings should be compatible with the data-driven approach of precision agriculture. This includes the development of coatings that respond to soil sensors or remote signals, or coatings embedded with indicators (e.g., color change, fluorescence) to indicate nutrient release status. While this may be more relevant for high-value crops, it represents a promising avenue for next-generation intelligent fertilizers.

### 8.6. Regulatory, Economic, and Farmer Adoption Aspects

Research must also extend beyond materials science to encompass regulatory compliance, safety evaluation, and economic feasibility. The development of cost-effective, farmer-friendly solutions, particularly for smallholder systems, requires simplified manufacturing, local feedstock availability, and compatibility with existing fertilizer infrastructure.

Starch-based CRFs stand at the cusp of scientific innovation and practical deployment. Addressing the outlined research gaps through multidisciplinary collaboration, field-scale testing, and sustainability assessment will be essential to unlock their full potential in climate-smart and resource-efficient agriculture.

Table 4 presents a concise summary of the key challenges encountered in the development and deployment of starch-based stimuli-responsive CRFs, along with targeted future directions aimed at overcoming these limitations.

## 9. Conclusions and Outlook

Stimuli-responsive starch-based biopolymer coatings are a polymer-engineered route to controlled-release fertilizers (CRFs) that couple nutrient delivery to soil cues (pH, moisture, temperature, ionic strength, and enzymatic activity). Starch’s abundance, biodegradability, and chemically tunable backbone enable coatings whose release rate is governed by defined mechanisms—ionization-driven swelling, osmotic counter-ion pressure, thermally driven coil–globule transitions, moisture-induced gel expansion, and enzyme-mediated chain scission—allowing spatial and temporal control that aligns with crop demand and field conditions.

This review consolidates recent advances in molecular design, fabrication, and performance evaluation of starch-based CRFs and synthesizes how specific structural modifications and stimulus–response mechanisms can enhance nutrient retention and soil health under realistic soil chemistries.

Despite significant progress, several challenges remain. Real-world deployment is limited by scalability issues, variability in field conditions, and the lack of long-term agronomic evaluations. The integration of multi-stimuli responsiveness, durable field stability, and compatibility with diverse soil microbiomes needs further investigation. Additionally, regulatory standards and cost-effectiveness must be addressed before widespread commercialization.

Limitations and trade-offs: Field performance can diverge from lab results due to variable soil chemistries and microbiomes: starch degradation and coating integrity depend on pH, salinity/ionic strength, moisture regimes, and enzyme activity, and may be altered by crosslinkers or inorganic fillers. Prolonging release can compete with rapid biodegradation, creating a design trade-off between release persistence and residue minimization. The cost and availability of modified starches and processing (e.g., grafting/crosslinking, fluidized-bed coating) may limit near-term adoption in low-input/smallholder systems unless manufacturing and dosing are optimized. These uncertainties motivate targeted field trials and techno-economic assessment alongside materials development.

To translate these materials into reliable field performance while addressing the above limitations, we identify the following near-term priorities:

- Designing multi-responsive and biodegradable systems tailored to specific soil types and crop cycles.
- Incorporating field-scale performance trials to validate lab-scale findings.
- Exploring synergistic interactions with beneficial soil microbes and root exudates.
- Using machine learning and modeling to predict release profiles under real-time conditions.
- Developing policy frameworks that support adoption of smart fertilizers in sustainable agriculture.
- By bridging materials science, agronomy, and environmental engineering, starch-based stimuli-responsive coatings can deliver context-specific benefits, for example, moisture-responsive hydrogels that swell during storm events and de-swell as soils dry to buffer rain-pulse leaching in rain-fed/semi-arid systems (see Section 3.3.3), and ionic-strength/pH-responsive matrices that maintain agronomically useful release profiles in alkaline–calcareous soils where Ca^2+^/Mg^2+^ and high salinity otherwise complicate nutrient management (see Section 3.3.1 and Section 3.3.6).

## Figures and Tables

**Table 1:** Summary of selected studies on starch-based smart coating systems for CRFs.

Ref	Material	Stimuli	Modification Method	Crop or Test Condition	Performance	Kinetic Model	Key Findings
							
[4]	Oxidized starch (OS)/PVA hydrogel (OSHU)	Moisture (swelling), biodegradation in soil	Oxidation of potato starch with NaClO; crosslinked with PVA	Soil incubation; pakchoi plant trial	34.6–57% urea released in 35 days; high swelling (2125.6%); water retention improved	First-order; Fickian diffusion	Pakchoi biomass improved; hydrogel showed excellent reusability, mechanical integrity, and nutrient release potential
							
[7]	Starch-g-GMA hydrogel + DMAAm + activated carbon (GMAAC)	pH, temperature, ionic strength	Grafting of glycidyl methacrylate (GMA) onto starch; incorporation of GMA-modified activated carbon (GMAAC)	No crop; tested for urea release and recyclability	Urea release sustained for >3 h; release only after matrix degradation; stable for ≥15 cycles	Not specified	Multi-responsive hydrogel with high swelling (438 g/g); mechanical stability and reusability confirmed; suitable for sustainable agriculture
							
[8]	Acrylamide-grafted starch hydrogel crosslinked with borax	Temperature	Free radical grafting using ceric ammonium nitrate (CAN); dynamic borate ester and H-bonding network	No specific crop; developed as material for smart agriculture and sensors	Exhibits temperature-responsive self-healing and mechanical recovery	Not applied	Demonstrated high mechanical strength and full self-repair within 12 h due to reversible borate ester formation; promising for smart agricultural carriers
							
[27]	Cassava starch hydrogel crosslinked with oxidized sucrose	Moisture (swelling), cytocompatibility	Central composite design (CCD); OS addition; crosslinking confirmed via FTIR and ^1^H NMR	Biomedical; no agricultural use or crop	95% swelling; WVTR = 714.9 g/m^2^·24 h; contact angle = 74.8°	None	Optimized for high tensile strength (27 MPa), high swelling, and excellent cytocompatibility (>100% cell viability); potential wound dressing application
							
[28]	Starch films reinforced with cellulose crystals or starch crystals	Environmental exposure (UV, thermal)	Melt blending with polysaccharide-based crystals	No crop—material characterization only	Improved Young’s modulus and tensile strength; UV shielding by starch crystal composite	None	Cellulose crystals enhanced thermal and mechanical performance; starch crystals improved UV shielding; fully edible and biodegradable films for packaging or capsule use
							
[31]	Modified potato starch with 5 wt% cellulose nanowhiskers (CNWs) or layered silicates (LSs)	Moisture, thermal (mechanical testing at various temperatures)	Plasticized with water and sorbitol; TEM used for structure assessment	No crop; mechanical and thermal characterization only	No fertilizer release tested	None (not a release study)	Nanocomposites showed significantly enhanced tensile and thermal properties; LSs gave higher storage modulus at elevated T; CNWs and LSs well dispersed via TEM
							
[36]	Starch/kraft lignin/graphene oxide (GO) bio-nanocomposite	Moisture (soil), mechanical resistance	Physical blending; GO added to enhance strength and barrier properties	Maize (Zea mays) in soil	Improved phosphorus retention; delayed release; increased crush resistance of coated TSP	Not specified	Coated TSP enhanced maize growth parameters; showed excellent structural integrity and sustainable release profile
							
[38]	Starch-g-poly(vinyl acetate) (St-g-PVAc) composite films	Water immersion and biodegradation in soil	In situ graft copolymerization of starch with vinyl acetate (VAc) using K_2_S_2_O_8_ initiator	No specific crop; studied in water and soil environments	Slow urea release: up to 28 h in water; films degraded in soil	Not specified	Grafting with hydrophobic PVAc reduced water swellability and improved release control; FTIR confirmed structural integrity; eco-friendly matrix for CRFs
							
[47]	Cassava starch–alginate–Ca^2+^ supramolecular hydrogel beads (s-PHBs)	pH, temperature, ionic strength, water hardness	Ca^2+^-induced crosslinking with phosphate encapsulation	Water and real rice field samples; no plant tested	Low initial burst, parabolic diffusion; low responsiveness to stimuli; stable release under extremes	Parabolic diffusion model	s-PHBs improved rigidity and stability; controlled P-release with high tolerance to environmental variation; commercial-scale potential as environmentally friendly CRFs
							
[58]	Starch–poly(acrylic acid) hydrogel crosslinked with Fe^3+^	Water degradation, biodegradation	Grafting and ionic crosslinking with Fe^3+^; 25% PAA in starch-based matrix	Soil burial for 60 days (no specific crop)	Extended nitrogen release due to denser membrane; 71.11% weight loss in 60 days	Not specified	Ionic crosslinking enhanced mechanical and thermal stability; biodegradable coating offers eco-friendly CRF alternative
							
							
[84]	Carboxymethyl starch sodium/sodium alginate/polydopamine composite	Water retention, photosensitive (light-triggered)	Crosslinking and surface coating in two-step method	Winter wheat under water stress conditions	Extended release over 30 days; photothermal conversion under light	Ritger–Peppas (best fit), Fickian diffusion	Excellent light-induced heating and water retention; SEM showed smooth coating; significantly improved wheat growth under drought
							
[68]	Starch-grafted polyacrylamide hydrogel encapsulating humic acid (HA)	Water-induced swelling, biodegradation	In situ radiation grafting of acrylamide onto starch; surface coating of HA with stearic acid/wax	Not crop-tested; release tested over 60 days	90% HA release in 53–60 days; initial burst reduced to ~30%	Not specified	Surface-coated HA prevented radical quenching; grafting efficiency up to 42.7%; performance met ISO/ASTM for SRFs; demonstrated structure–release relationship
							
[91]	Dialdehyde cassava starch/sodium alginate composite hydrogel beads	pH, redox	Covalent crosslinking with cystamine and ionic crosslinking with Ca^2+^	Aqueous and simulated soil medium with DIN pesticide	Dual-stimuli-responsive release; higher DIN release at neutral/alkaline pH and under reducing conditions	Korsmeyer–Peppas	Environmentally friendly formulation for targeted, sustained pesticide release using a green dual-responsive system
							
[72]	Cassava starch-based biodegradable composite films	Biodegradation (soil microbes)	Box–Behnken statistical design to optimize formulation	Indoor soil burial (no crop) for 28 days	Degradation rate linked to microbial activity, water absorption, and film structure	Second-order polynomial (RSM)	Weight loss and SEM confirmed microbial degradation; films suitable for landfill disposal due to limited lifetime in soil
							
[73]	Corn starch–sodium alginate liquid mulch film	Biodegradation (soil microbial)	Moist heat modification; blended with 50% glycerol and 4% citric acid	Soil burial at 25 °C for 25 days (no specific crop)	Fully degraded macroscopically by day 25; increased soil organic matter	Microbial community succession analysis (no drug-release kinetics)	Degradation altered bacterial diversity and reduced abundance; film had improved mechanical integrity via H-bonded network among –COOH and –OH groups
							
[75]	Starch–KNO_3_ microspheres embedded in PVA matrix	Biodegradation (microbial)	Spray drying of starch microspheres; melt extrusion with PVA	100-day soil burial test; microbial activity tracking	Gradual nutrient and polymer degradation; increased CO_2_ and fungal growth over time	Not applied	Starch promoted microbial adhesion and enhanced biodegradation of the PVA–starch fertilizer matrix, supporting sustainable nutrient delivery and soil health
							
[67]	Microalgae–chitosan–starch hydrogel	Moisture/swelling	Crosslinked hydrogel with encapsulated urea; physical blending with microalgae	Soil-blended test (no specific plant)	78–95% urea released in 30 days; loading up to 440%	Freundlich (loading), Zero-order, Higuchi, Korsmeyer–Peppas (release)	Excellent water retention and re-swelling in soil; potential soil conditioner; enhanced WHC with only 1 g hydrogel per 50 g soil
							
[79]	Starch-based hydrogel (HG) for water retention	Ionic strength, pH	Not chemically modified; tested in dry/swollen/sandwich forms	Tested in sandy and clayey soils; no crop	Up to 62 mL water/g HG in sandy soil; no quantity dependence in clay	Not specified	Swollen/sandwich application was less effective; dry application best; water absorption 5× higher in low ionic strength; effective at pH 5 and 7
							
[91]	Starch/polyvinyl alcohol blend film crosslinked with formaldehyde	Water immersion, biodegradation in soil	Blended at 7:3 (starch:PVA) and chemically crosslinked using 10 wt% formaldehyde	No specific crop; material tested in soil for biodegradability	Smooth, dense crosslinked membrane reduced release rate of urea	Not specified	Crosslinking enhanced compatibility and reduced porosity; FTIR and XRD confirmed chemical bonding and structure; film biodegraded in soil with controlled nutrient release
							
[92]	Tapioca starch modified with PVA and citric acid; outer geopolymer layer	Moisture	Crosslinking (citric acid), plasticizing (PVA), dual-layer coating via rotary fluidized bed	Laboratory-scale fluidized bed; no specific crop	Extended N release up to 22 days; R^2^ > 0.99 for optimal model	Not explicitly reported; focus on empirical release and optimization	Process optimized using RSM and GA; double coating significantly delayed nitrogen release
							
[93]	Starch/PBAT composite film-coated urea	Water exposure (hydrolytic degradation)	Melt blending and film compression with PBAT and glycerol; no solvents	Rice field trials and water incubation at 25 °C and 40 °C	Tunable nutrient release from 20 to 100 days depending on film thickness and PBAT ratio	Prediction model (RSM), no standard kinetic model	Solvent-free biodegradable CRF films with excellent water resistance and low material usage (1–2%); validated by CT imaging and rice yield improvement
							
[95]	Sago starch films with red cabbage anthocyanins (RCA) (8–16% *w*/*v*)	pH, temperature	Solvent casting with glycerol plasticizer	Food simulants: 3% acetic acid and 10% ethanol; no crop	Lower anthocyanin release at room temp than fridge temp; RCA reduced crystallinity and increased WVP	None specified	Indicator films showed color change with spoilage simulants; 16% RCA film had 74% lower crystallinity and 0.6% less release at room temp; simple smart packaging tool
							
[96]	Nanoclay-reinforced starch-grafted poly[(acrylic acid)-co-acrylamide] hydrogel composites	Soil type (acidic vs. neutral), water	Graft polymerization of acrylic acid and acrylamide onto wheat/maize starch with nanoclay integration	Soil incubation under Assam and Delhi soil conditions	Maize starch-based NCPCs showed slowest N release (~62–65% in 30 days)	Not specified	Maize starch grafts performed best in acidic soil; FTIR and XRD confirmed structural reinforcement; nanoclay reduced burst release and improved water retention
							
[97]	Cassava starch/polyacrylamide/natural rubber + montmorillonite (0–10 wt%)	Moisture, biodegradability	Free-radical polymerization + semi-IPN + glutaraldehyde crosslinking	Field soil, urea encapsulation in wax-coated hydrogel	BHM3 (3% MMT) showed best release: high swelling (7074%), 58% biodegradation, sustained release > 35 days	Not specified	BHM3 improved N release by 39.1%, mechanical strength by 260%; low-cost SRF alternative with field growth success; best bio-safety and yield profile among formulations
							
[98]	Thermoplastic starch films with 5–10 wt% cellulose nanofibers from rice straw	Moisture (humidity), mechanical stress	Chemo-mechanical extraction of CNF + film casting + ultrasonication	No crop tested; mechanical and moisture barrier testing only	Not tested for fertilizer release	None	CNFs enhanced yield strength, Young’s modulus, and humidity resistance; glass transition temp increased with CNF content; reduced transparency with higher CNF; good fiber dispersion seen in SEM
							
[99]	Nanozeolite–chitosan/sago starch (NZ–CS/ST) composite	Moisture/swelling	Ionotropic gelation with sodium tripolyphosphate; NZ prepared by co-precipitation + annealing	Philodendron sp. pot experiment	41.93% urea and 64.0% P released in 14 days; improved water retention	Not specified	Superior growth over control and urea-only group; enhanced swelling with higher MW chitosan and crosslinking time
							
[100]	ST–PHEMA–GO hydrogels (starch, 2-hydroxyethyl methacrylate, graphene oxide)	pH-responsive swelling	Gamma-radiation induced polymerization; GO ratios varied	Not crop-tested; general release system studied	Swelling varied with pH (3, 7, 11), GO content, and irradiation dose; Fickian diffusion observed	Korsmeyer–Peppas (*n* < 0.5)	High mechanical stability and tunable swelling; potential for sustained release in various environments
							
[101]	Double-coated urea: Inner EC, outer starch-based SAP (from maize, potato, and pea starches)	Moisture-responsive (water absorption and swelling)	Twin-roll mixer to synthesize starch-based SAPs; inner EC coating applied	Soil incubation studies (no crop tested)	Steady nitrogen release for >96 h; potato starch-SAP most effective in prolonging release	Not explicitly stated	Potato starch-based SAP significantly improved water retention and slowed nutrient release under soil conditions
							
[102]	Starch microspheres encapsulating Fe, Cu, Mn	Moisture/swelling	Spray-dried gelatinized starch with micronutrients (monoelemental dispersions)	Water-based nutrient release (no crop)	Larger particles swelled more and released nutrients more slowly	Peppas-Sahlin model	Swelling delayed micronutrient release; different metals altered morphology, swelling, and release kinetics
							
[103]	Porous carboxymethyl starch (PCS) + Fe-P complex, zein coating	Moisture	Phosphorus and Fe loaded onto PCS; coated with zein	Soybean; pot experiment	18% phosphorus released in 30 days; sustained release	Isotherm and kinetic studies applied	Phosphorus use efficiency increased to 68%, ~3× higher than conventional phosphate fertilizer; Fe acts as a bridge and nutrient source
							
[104]	Starch-g-poly(acrylic acid-co-acrylamide) with natural char nanoparticles (NCNPs)	pH, ionic strength	Reinforcement with NCNPs as nano-filler and crosslinker	Soil column test with urea leaching	70% nitrogen released in 21 days; enhanced retention in basic and neutral pH	Not explicitly modeled	NCNPs doubled water retention and reduced nitrate leaching from 591.8 to 49.5 mg/L; enhanced degradation and nutrient efficiency
							
[105]	Cassava starch-g-poly(acrylic acid)/natural rubber/PVA semi-IPN hydrogel	pH, temperature, ionic strength	Grafting PAA onto starch, blended with NR and PVA, coated over urea with wax layer	Soil and water; chili plant test	47.5% release in water (168 h), 38.5% in soil (30 d); best for NR:PVA 9:1	Korsmeyer–Peppas	Low-cost biodegradable formulation; sustained nutrient release; responsive to environmental triggers; improved chili growth
							
[106]	Zeolite-coated urea using corn starch as binder	Water exposure (leaching)	Pan granulation with five binders including starch	30-day soil column study for UZ-AP vs. uncoated urea	Acrylic polymer gave best performance; starch-bonded coatings less stable with faster N release	Not specified	Corn starch served as a natural binder for zeolite coating, but had lower crushing strength and faster N release vs. synthetic binders; zeolite + binder system reduced leaching by 65% (UZ-AP)
							
[107]	Cassava starch-g-PAA/NR/PVA hydrogel	Moisture, biodegradation	Grafting with acrylic acid, semi-IPN with NR/PVA, crosslinked using GA, MBA, or EGDMA	Maize plant growth performance and soil tests	67.7–78.3% release in 30 days; best for GA (UCSBw-G2); 20.2% WHC retained at 30 days	Not explicitly mentioned	GA-crosslinked hydrogel showed highest water retention, best degradation rate (1.37%/day), improved biosafety and plant performance compared to other variants and commercial urea
							
[108]	Modified starch binder (MSB) blended with pyroligneous acid and bio-oil in biochar-based fertilizer	Moisture	MSB derived via chemical modification; combined with PA and BO as binders	No plant growth test; characterization + nutrient leaching	PA + BO + MSB combination slowed P release by 4.7–21.2% compared to individual binders	Ritger–Peppas model (best fit)	Triple-binder system improved structural integrity and economic efficiency; MSB viscosity crucial for NPK retention
							
[109]	Cassava starch with alkyd resin (from castor and rubber seed oils)	pH, Time	Alkyd resins modified with sorbitol, maleic/phthalic anhydride; blended with starch	No plant used; urea release tested at pH 8 over 24 days	31.66–48.61% N release depending on coating thickness	First-order and Korsmeyer–Peppas models	SEM and FTIR confirmed compact coatings; release rates met EN 13266 standard for CRFs
							
[110]	Cassava starch + cassava bagasse composite	Soil moisture	Physical blending with up to 50 wt% urea; plasticized films	Soil incubation (no specific crop); 15 days	95% urea release in 15 days for films with 50 wt% urea	Not explicitly stated	Urea improved flexibility and biodegradability; 57% weight loss in 15 days; suitable for eco-friendly fertilizer delivery
							
[111]	Sodium carboxymethyl starch (SCS) with polydopamine	Moisture	Chelation with Fe ions; coating with Pdop and SCS for macro/micronutrient integration	Corn field test (multi-nutrient delivery)	Zn, P, Fe reached 60% release in 30 days; ~60% nutrient use efficiency	Not specified	Novel coating system improves macronutrient uptake and micronutrient efficiency (e.g., Fe chelation); environmentally friendly approach
							
[112]	Sulfonated corn starch/poly(acrylic acid) with phosphate rock	Moisture	Synthesis of a superabsorbent (SCS/PAA) embedding phosphate rock (PHR)	Soil incubation (no specific crop)	Max swelling: 498 g/g (H_2_O), 65 g/g (NaCl); slow P and K release confirmed via experiments	Not specified	Integrated water retention with nutrient delivery; SCS improved P release from PHR matrix
							
[113]	Starch acetate/glycerol/polyvinyl alcohol (SA/GLY/PVA) biocomposites	Moisture	SA grafted with GLY and PVA to form multilayer hydrophobic coatings on DAP granules	Water-based nutrient release tests (no crop)	Double-layer coating delayed nitrogen release 3.2× compared to uncoated DAP	Not specified	70% SA-based coatings formed smooth cohesive films with strong adhesion, reducing burst release of N and P
							
[114]	PVA–starch formulation with Cu–Zn/CNF nanocomposite	Moisture/biodegradation	In situ dispersion of Cu–Zn-loaded carbon nanofibers (CNFs) in PVA–starch during polymerization	Chickpea plant growth in garden soil	Gradual release of Cu and Zn; 5.3 ± 0.05 mg/g Cu and 2.8 ± 0.1 mg/g Zn; enhanced growth and reduced ROS	Not explicitly modeled	PVA–starch matrix enabled micronutrient-controlled release; improved plant height, ROS scavenging, and nutrient translocation
							
[115]	Starch + maleic-anhydride-modified β-cyclodextrin + acid-treated halloysite nanotubes	Moisture, ionic strength	Free radical copolymerization with acrylic acid and acrylamide	Water and soil conditions; no specific crop	Slower release in soil than in water; high water retention; release fitted Fickian diffusion in soil	Fickian diffusion model	Halloysite enhanced urea loading and controlled release; excellent swelling and retention properties for agro-applications
							
[116]	High-amylose or waxy starch grafted with acrylamide and crosslinked with N,N′-MBA	Moisture, water absorption	One-step reactive melt-mixing with ceric ammonium nitrate (CAN) initiator and NaOH saponification	Urea release in water (no specific crop)	Urea release rate influenced by gel strength and microstructure; slower release from stronger high-amylose gel networks	Not explicitly stated	Crosslinked high-amylose gels showed higher storage modulus, smaller pore size, and controlled nutrient release

**Table 2:** Summary of evaluation aspects, methods used, and corresponding purpose in assessing stimuli-responsive starch-based coatings.

Evaluation Aspect	Methods Used	Purpose
Morphology	SEM, AFM	Surface uniformity, coating thickness
		
Thermal Behavior	DSC, TGA	Stability of coating
		
Chemical Structure	FTIR, XRD	Functional groups, crystallinity
		
Stimuli Response	Swelling, LCST, pH-responsiveness	Triggered release
		
Release Performance	Kinetic modeling	Rate and mechanism of release
		
Agronomic Effectiveness	Pot/field trials	Real-soil validation

**Table 3:** Comparative matrix of starch and selected biopolymers for CRF and related applications.

Parameter	Starch	Chitosan	Cellulose	Alginate
Source and Availability	Abundant, derived from cereals, tubers	Obtained from chitin in crustacean shells	Widely available from plant biomass	Extracted from brown seaweed
Biodegradability	High, enzymatically degradable	High, enzymatically degradable	High, slow degradation in some soils	High, ionically and enzymatically degradable
Cost-effectiveness	Very low cost	Moderate cost	Low–moderate cost	Moderate cost
Mechanical Properties	Moderate strength; can be improved via crosslinking/nanofillers	Good film-forming, moderate tensile strength	High tensile strength, low flexibility	Flexible gels, low tensile strength
Functional Groups for Nutrient Binding	Hydroxyl groups; modifiable to introduce carboxyl/amine	Amino groups (cationic), hydroxyl groups	Hydroxyl groups	Carboxylate groups (anionic)
				
Responsive Capabilities	pH, moisture, temperature, ionic strength, enzymatic	pH, moisture, temperature, enzymatic	Limited pH/moisture response	pH, ionic strength, moisture
Representative Applications	CRF coatings, drug delivery, biodegradable packaging	CRF coatings, antimicrobial films, drug delivery	Composite CRF films, reinforcement in bioplastics	Encapsulation of fertilizers, biomedical gels

**Table 4:** Key challenges and future directions in the development and deployment of starch-based stimuli-responsive CRFs.

Challenges	Future Directions
	
High formulation cost	Low-cost, bio-derived polymers and green synthesis
	
Variable soil conditions (pH, enzymes, moisture)	Soil-specific smart coating customization
	
Uncertainty in long-term biodegradability	Eco-safety and residue toxicity studies
	
Limited field performance data	Field validation under diverse agro-ecosystems
	
Weak regulatory frameworks	Integration of machine learning and modeling tools
	

## Data Availability

No new data were created or analyzed in this study.
